# On the fast track: hybrids adapt more rapidly than parental populations in a novel environment

**DOI:** 10.1093/evlett/qrad002

**Published:** 2023-03-01

**Authors:** Jonna Kulmuni, Bryn Wiley, Sarah P Otto

**Affiliations:** Organismal & Evolutionary Biology Research Programme, University of Helsinki, Helsinki, Finland; Tvärminne Zoological Station, University of Helsinki, Hanko, Finland; Institute for Biodiversity and Ecosystem Dynamics, Department of Evolutionary and Population Biology, University of Amsterdam, Amsterdam, The Netherlands; Department of Zoology and Biodiversity Research Center, University of British Columbia, Vancouver, Canada; Department of Zoology and Biodiversity Research Center, University of British Columbia, Vancouver, Canada

**Keywords:** hybridization, adaptation, climate change, polygenic trait, Fisher’s geometric model

## Abstract

Rates of hybridization are predicted to increase due to climate change and human activity that cause redistribution of species and bring previously isolated populations into contact. At the same time climate change leads to rapid changes in the environment, requiring populations to adapt rapidly in order to survive. A few empirical cases suggest hybridization can facilitate adaptation despite its potential for incompatibilities and deleterious fitness consequences. Here we use simulations and Fisher’s Geometric model to evaluate the conditions and time frame of adaptation via hybridization in both diploids and haplodiploids. We find that hybrids adapt faster to new environments compared to parental populations in nearly all simulated scenarios, generating a fitness advantage that can offset intrinsic incompatibilities and last for tens of generations, regardless of whether the population was diploid or haplodiploid. Our results highlight the creative role of hybridization and suggest that hybridization may help contemporary populations adapt to the changing climate. However, adaptation by hybrids may well happen at the cost of reduced biodiversity, if previously isolated lineages collapse into one.

## Introduction

Human-caused environmental change, with broad-scale habitat alteration, spread of invasive species, and accumulating pollutants, imposes altered selection pressures on species across the globe (reviewed by Hendry et al., 2017; Otto, 2018). Climate change is also an increasing threat to biodiversity (Urban, 2015), as species face rising mean temperatures, ocean acidification, and more variable weather conditions (IPCC, 2022). In extreme cases, these changes require rapid adaptation for natural populations to avoid extinction (Bell, 2017). However, adaptation via new mutations may be too slow in many taxa, and standing genetic variation might not be sufficient to help small, isolated populations. By combining genetic variation from two parental lineages, hybridization can supply the raw material for natural selection to fast-track adaptation (Grant & Grant, 2019; Hamilton & Miller, 2016; Hedrick, 2013). In contrast to standing genetic variation or de novo mutations, hybridization between populations with a history of adaptation instantaneously increases genetic variation at numerous loci with variants already proven functional in another genomic background. Rates of hybridization have increased due to human-mediated redistribution of species (Ottenburghs, 2021) and are predicted to increase due to climate change, as shifts in species’ ranges bring previously isolated populations into contact (Chunco, 2014; Scheffers et al., 2016). However, novel combinations of divergent alleles can also lead to incompatibilities and lowered fitness (Coyne & Orr, 2004). Although empirical reports on the adaptive potential of hybridization exist as described in the discussion (e.g., Martin-Roy et al., 2021; Meier et al., 2017; Mitchell et al., 2019; Stelkens et al., 2014b), systematic evaluation of its adaptive value for polygenic traits is lacking. Moreover, when and over what time frame hybridization is predicted to facilitate rapid adaptation has not been systematically evaluated in models of evolution.

Here we use simulations and Fisher’s geometric modeling framework to compare hybrid and parental populations in their speed of adaptation when facing a novel environment. We predict that hybrids should adapt faster compared to parental populations due to increased genetic variation in hybrids compared to parents, an advantage that can offset and even reverse reduced hybrid fitness due to intrinsic incompatibilities or disruption of local adaptation. Indeed, there is increasing empirical evidence of introgression following hybridization in a wide variety of animals and plants (Edelman & Mallet, 2021; Hedrick, 2013; Suarez-gonzalez et al., 2018).

We also compare the speed of adaptation in diploid and haplodiploid populations. In haplodiploid organisms (e.g., ants, bees, thrips, some beetles) one sex is haploid and another sex (females) is diploid. Over 15% of Arthropod species are haplodiploid, representing a significant proportion of animal biodiversity (de la Filia et al., 2015). Furthermore, sex chromosomes of diploid organisms are essentially haplodiploid and many organisms have a haploid stage (e.g., pollen, sperm, eggs, etc.). This means that many organisms experience haploid selection in addition to diploid selection. Selection operates differently in haploid compared to diploid genomes, because new alleles that are less than fully dominant experience stronger selection in haploids (Nouhaud et al., 2020). Consequently, beneficial alleles are more strongly favored, and deleterious alleles more readily purged, in haploid compared to diploid genomes. On the other hand, genetic variation can be more easily maintained in diploids when heterozygotes have high fitness, which is common in the framework of Fisher’s geometric model (Sellis et al., 2011). We explore the net result, comparing the speed of adaptation in diploid and haplodiploid hybrids.

We chose Fisher’s geometric model as a framework for our study. Mutations in this model each affect a number of phenotypic traits (*n*), bringing an individual either closer to or further from a single fitness peak. In addition to including pleiotropic effects of mutations on multiple traits, Fisher’s geometric model also naturally incorporates epistasis, because the fitness effect of a mutation depends on the other alleles carried in that genome (i.e., the genomic background). This means a particular mutation may be beneficial in one individual but deleterious in another. Similarly, environmental change can be modeled as a shift in the optimum, changing selection on each allele. This is in contrast to the “traditional” population genomic framework, where mutations themselves are assigned selection coefficients and where epistasis between pairs of loci is fixed. Fisher’s geometric model has been used as a framework to study adaptation to a changing environment (e.g., Schneemann et al., 2020; Thompson et al., 2019; Yamaguchi & Otto, 2020) and for analysis of hybrid fitness, for which this model encompasses many aspects of real data, like heterosis and incompatibilities in hybrids (Fraïsse et al., 2016; Schneemann et al., 2020; Simon et al., 2018b; Yamaguchi & Otto, 2020). In the main text, we model evolution with five continuously varying phenotypic axes (*n* = 5), with environmental change altering the position of the fitness optimum. As an example, one trait axis could represent thermal tolerance and another one feeding rate.

Our simulations show that hybrids adapt faster than parents to novel environments in nearly all scenarios considered. The only scenario where hybrid and parental fitnesses are comparable is when the novel environment is close to the environment in which parents were adapted to. In previous literature, the deleterious consequences of hybridization are emphasized, because hybrid fitness is typically measured in one or both parental environments where parents are fit and hybrids unfit. However, upon rapid environmental change, like that under climate change, the parental environment no longer exists and both parents and hybrids need to adapt to the novel environment. In this case hybrids are able to take a fast track to adaptation due to the higher degree of genetic variation they harbor. We discuss our results in the context of a changing climate, but simulated scenarios could fit equally well to other rapid environmental changes.

## Methods

To study adaptation to novel environments we used simulations in SLiM 3.6 (Haller & Messer, 2019). In short, we used the framework of the Fisher’s Geometric Model as in Yamaguchi and Otto (2020) but with individual based simulations. We set the fitness landscape to contain five trait axes and the fitness optimum to be defined in relation to these axes. We assume an initial fitness optimum at {0,0,0,0,0} (all traits in the five dimensional space are at the origin), with the phenotype of all individuals initially at this optimum and no genetic variation. We then alter the environment by shifting the optimum (e.g., to {1,0,0,0,0} where an increase in the first trait by 1 is favored) and track adaptation as an increase in mean fitness over time. We simulated a single chromosome of one megabase with a mutation rate of 10^−8^ per site and scaled the number of individuals within a population (N_pop_) and recombination rate so that haplodiploids (N_pop_ = 2000, sex ratio 50:50, recombination rate per basepair = 10^−6^) and diploids (N_pop_ = 1500, sex ratio 50:50, recombination rate = ⅔ 10^−6^) have comparable effective sizes and recombination rates (as in Bendall et al., 2022). We adapted the haplodiploid SLiM model from Pracana et al. (2022). Each mutation has an additive effect on the phenotype (codominant, but see Supplementary Figures 2 and 3). Nevertheless, dominance and epistasis emerge depending on the fitness of those phenotypes. Specifically, we assumed fitness was Gaussian in shape, dropping from a height of one for phenotypes at the optimum to *exp*(−*x*^2^) for phenotypes that are a Euclidean distance of *x* away from the optimum (i.e., *q* = 1 in Equation (1) of Yamaguchi & Otto, 2020). New mutations point in a random direction in the phenotypic space, with an effect size drawn from an exponential distribution with mean λ = 0.2 when fully expressed (i.e., in haploids or homozygous diploids). In Supplementary Material, we investigate the robustness of our results to alternative assumptions in different adaptational scenarios (Supplementary Figure 1), changing the dominance of new mutations (Supplementary Figures 2 and 3), the mean effect of mutations, λ (Supplementary Figure 4), the number of dimensions, *n* (Supplementary Figures 5 and 6), the shape of the fitness surface, *k* (Supplementary Figures 7 and 8), and ploidy level (considering tetraploids in Supplementary Figure 9).

**Figure 1.**
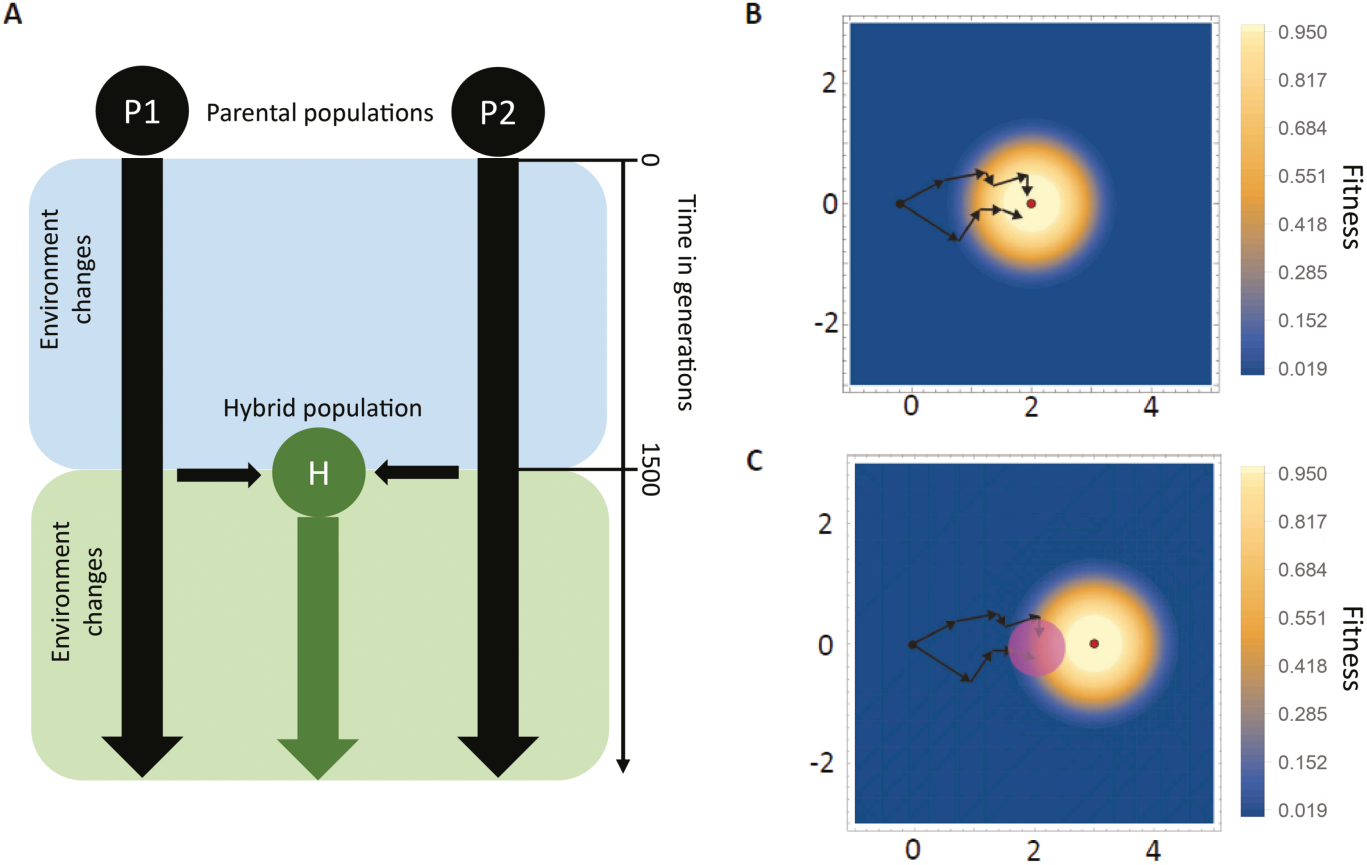
Simulation set up. We used SLiM and Fisher’s Geometric model to simulate two allopatric parental populations adapting to either the same or different environments for either a diploid or haplodiploid species (panel A). In Fisher’s geometric model, populations accumulate mutations that have an additive effect on phenotype. A hypothetical example is shown in B, where two parental populations accumulate mutations (arrows) that move the average phenotype from the origin (black dot) to an environmental optimum (red dot) at {2,0} in a two-dimensional trait space. After parental populations are adapted, a hybrid population with 50:50 ancestries is created (generation 1500), but populations experience no further gene flow during the simulation. All three populations (P1, P2, H) face a new environment, and we track the speed of adaptation by quantifying the mean fitnesses of males and females in every generation. A hypothetical example of this is depicted in C, showing the optimum shift to {3,0} (red dot) and the creation of a hybrid population (purple circle). In our simulations, we use *n* = 5 trait dimensions and allow for more genetic variation not able to be shown in these hypothetical examples.

The evolutionary history of parental populations has been shown to impact hybrid fitness (Barton, 2001; Schneemann et al., 2020; Simon et al., 2018a; Yamaguchi & Otto, 2020), and here we explore its impact on hybrid adaptation. To vary evolutionary history, our simulations track two allopatric parental populations that are initially genetically identical with an optimum at the origin at generation 0. The parental populations then adapt for 1500 generations either to identical or divergent environments. After parental populations (P1 and P2) are adapted, a hybrid population (H) is created at generation 1500, with no further gene flow. Generation 1500 was chosen to ensure parental populations are well adapted across the different simulation scenarios. The hybrid population initially consists of N_pop_/2 descendants of P1 and N_pop_/2 descendants of P2. All three populations (P1, P2, H) then face a novel environment starting at generation 1500, and we compare the speed of adaptation by quantifying the mean fitness of males and females within a population every generation (Figure 1). Genetic incompatibilities naturally arise in Fisher’s geometric model (see below), but we also consider additional intrinsic genetic incompatibilities (Bateson-Dobzhansky-Muller Incompatibilities, “BDMIs”) that do not depend on the environment in Supplementary Figures 10–13. In the main text, all populations are kept at constant size, but Supplementary Figures 14–18 and Supplementary Table 1 allow for the possibility of demographic changes and even extinction as a result of the environmental shifts. Results are a mean over 100 simulation replicates. Simulation scripts are available from Dryad (Dryad DOI https://doi.org/10.5061/dryad.vhhmgqnz5).

## Results

### Hybrids adapt faster to new environments compared to parental species

**Figure 2.**
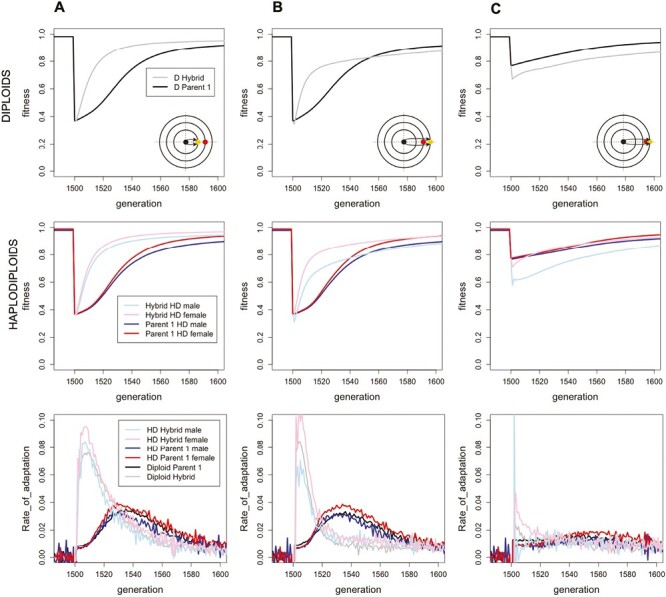
Adaptation in hybrid and parental populations when environment changes along one trait dimension. All parental populations experience an environmental shift along the first trait axis (first row diploids, second row haplodiploids). Schematic inset figures depict the simulation design. At generation 0, the optimum moves from {0,0,0,0,0} (black point in the inset) to a new position along the first trait axis (yellow dot in the inset), with the two black arrows depicting the first phase of parental adaptation. The optimum then moves again at generation 1500 (red dot), at which point a hybrid population is created and its rate of adaptation compared to the parental populations (bottom row; measured as the change in mean fitness from one generation to the next relative to how far the mean fitness currently is from the optimum: $\Delta \underline{W}(t)/(1-\underline{W}(t))$). (A) Parents adapt first to an optimum at {1,0,0,0,0}, which then shifts at generation 1500 to {2,0,0,0,0} for all populations. (B) Parents adapt first to an optimum at {3,0,0,0,0}, which then shifts to {2,0,0,0,0}. (C) Parents adapt first to an optimum {3,0,0,0,0}, which then shifts to {2.5,0,0,0,0}. Male and female fitnesses are equal in diploid populations but differ in haplodiploid populations.

**Figure 3.**
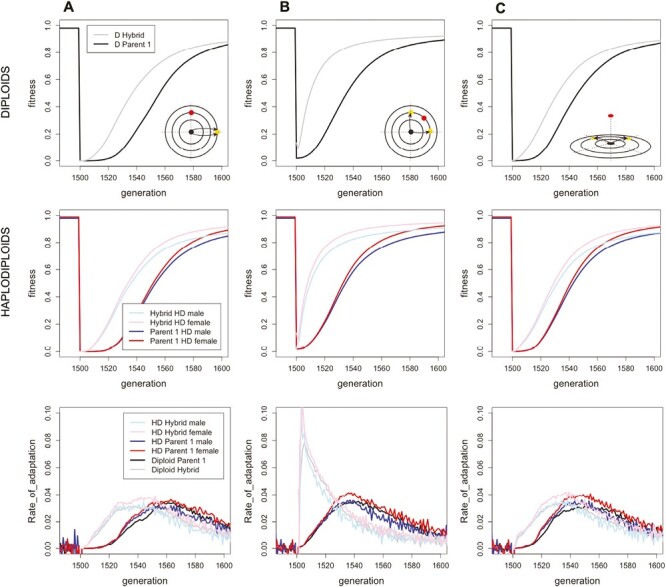
Adaptation in hybrid and parental populations when the environment changes along different trait dimensions. The figure is similar to Figure 2 except that different scenarios of environmental changes are considered and parental adaptation is not parallel in B and C. (A) Parents adapt first to an optimum at {3,0,0,0,0}, which then shifts at generation 1500 to {0,2,0,0,0}. (B) Parental populations 1 and 2 adapt to different optima in the first 1500 generations ({2,0,0,0,0} and {0,2,0,0,0}, respectively for the two parental populations), after which the optimum moves to {2,2,0,0,0} for all populations. (C) Parental populations 1 and 2 adapt to different optima in the first 1500 generations ({2,0,0,0,0} and {0,2,0,0,0}, respectively), after which the optimum moves to {0,0,2,0,0}.

Because different mutations arise and fix in isolated parental populations, genetic incompatibilities naturally emerge in Fisher’s geometric model. When combined together in hybrids, these mutations can over- or undershoot the optimum and/or break apart compensatory changes along other phenotypic axes, resulting in hybrid breakdown (Yamaguchi & Otto, 2020). In most of our simulations where the optimum shifts after hybridization, however, these incompatibilities have a relatively small impact on fitness compared to the effect of the new environment, so that initially parental and hybrid populations are nearly equally maladapted (compare fitnesses at generation 1500 in Figures 2 and 3). Only when the new environment is similar to the parental environment (e.g., Figure 2C) do we see substantially reduced initial hybrid fitness relative to the parental populations, reflecting the accumulation of genetic incompatibilities between the parents.

Regardless of these initial differences in fitness, however, hybrid populations adapt faster than the parental populations to all environments explored (Figures 2 and 3). As a result, fitness in a novel environment rises rapidly tens of generations faster in hybrids than in parental populations in most scenarios explored. Hybrids adapt faster than parents whether the environment changes along the same axis (Figure 2) or along a different axis (Figure 3), and whether the two parental populations adapt in parallel or not. Only when the novel environment is similar to that experienced by the parents is the faster rate of hybrid adaptation too modest and the initial incompatibilities too great for hybrids to outperform the parental populations (Figure 2C). Interestingly, Figure 2B illustrates an intermediate case where hybrids adapt more rapidly to the novel environment, initially raising mean fitness above the parents, but this advantage eventually reverses, suggesting that residual incompatibilities can eventually hamper hybrid populations if the parental populations are also able to adapt.

**Figure 4.**
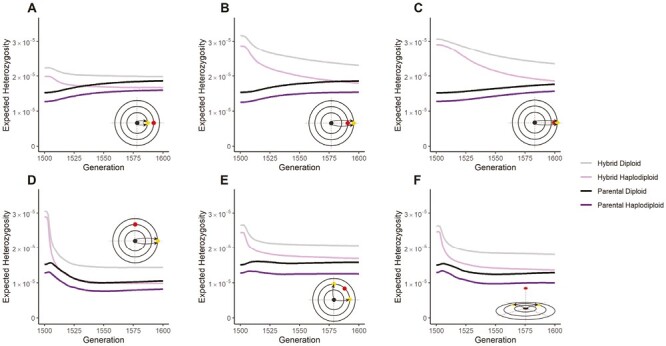
The expected heterozygosity (π) in hybrid and parental haplodiploid and diploid populations. The simulation scenarios are the same as illustrated in Figures 2 and 3, with the y-axis showing the expected heterozygosity, i.e., the probability that two randomly chosen alleles from the population are different at a site, averaged over all one million sites in the genome.

As confirmed in Figure 4, hybrids initially harbor more genetic variation than their parental populations, explaining the elevated rate of adaptation, but this elevated diversity decays as adaptation proceeds and beneficial alleles fix. The speed of adaptation and the relative benefits of hybridization depend also on the effect size of the mutations. When mutations have a larger effect size, on average, parental populations adapt faster due to the increased mutational variance and the difference between parents and hybrids in their speed of adaptation diminishes (Supplementary Figure 4).

The above simulations assume that mutations have additive effects on phenotype (*h* = 0.5), with dominance in fitness emerging from the fitnesses of homozygous and heterozygous individuals given the optimal fitness in the current environment. In Supplementary Materials, we also consider cases where new mutations are always partially recessive (*h* = 0.2) or dominant (*h* = 0.8) on a phenotypic scale (Supplementary Figures 2 and 3). Hybrids continue to adapt faster than parental populations, although the differences are more modest when mutations are more dominant (e.g., Supplementary Figure 2D). Dominance on a phenotypic scale increases the chance that new beneficial mutations fix and reduces the benefits of the higher initial genetic variation resulting from hybridization.

As explored in Supplementary Materials, the observation that hybrids adapt faster than parental populations when facing a new environment is robust to changes in the number of trait dimensions, *n* (Supplementary Figures 5 and 6), the shape of the fitness surface (Supplementary Figures 7 and 8), the inclusion of intrinsic BDMIs (Supplementary Figures 10–13), and explicit demography allowing population size to change (Supplementary Figures 14–18). Interestingly, this higher rate of adaptation even rescued hybrid populations from extinction more often than parental populations when the risk of extinction was high (Supplementary Figure 16). However, as more numerous intrinsic incompatibilities accumulate, hybrids have less access to the benefits of faster adaptation in many BDMI scenarios due to stronger initial costs of hybridization.

### Hybrid haplodiploid and diploid populations adapt at comparable rates

We also considered whether ploidy level impacts the speed of adaptation for hybrids relative to parental populations. Of particular interest was whether animals that are haplodiploid (e.g., hymenopteran pollinators) would be better or less able to adapt to a novel environment than an equivalent diploid species. Our initial hypothesis was that haploidy would increase the efficiency of selection, with male haploids expressing the full phenotypic effect of mutations and allowing haplodiploids to adapt faster than diploid populations. In particular, theory predicts that the fixation probability,*P*, for a new mutation with small selective benefit, *s*, is higher in haplodiploid populations ($P=\frac{1}{3}2s+\frac{2}{3}2hs$; (Charlesworth et al., 1987) than in diploid populations (P=2hs; (Haldane, 1927). However, similar patterns of adaptation were observed in haplodiploids and in diploids (Figures 2 and 3, Supplementary Figures 2 and 3), with hybrids adapting faster than parental populations regardless of ploidy level. Recall that we scaled our simulations so that haplodiploids and diploids would have the same effective population sizes and recombination rates, helping to explain why similar patterns were observed. Furthermore, selection in our simulations was very strong following environmental change, reducing the difference that ploidy makes to the fixation probability of beneficial mutations (*P* approaches one as *s* becomes large for all populations, both diploid and haplodiploid).

In addition, we find that diploids harbor more genetic diversity compared to haplodiploids both within hybrid and parental populations (Figure 4). In part, this pattern reflects more efficient purging of deleterious mutations in haplodiploid populations than in diploid populations, leading to lower initial levels of standing genetic variation in the parental haplodiploid populations, as well as over the time course of adaptation. Another contributing factor is that evolution within Fisher’s geometric model induces heterozygous advantage in diploids whenever the phenotypic effect of a mutation straddles an optimum (Sellis et al., 2011), allowing more genetic variation to be maintained in diploid than in haplodiploid populations.

Interestingly, haploid males always have a lower fitness compared to diploid females in haplodiploid populations, even when the populations are at their fitness optimum and well adapted (Figures 2 and 3). This is due to the fact that in Fisher’s geometric model, the haploid males are more likely to overshoot the fitness optimum. By contrast, diploid females have twice as many alleles contributing to the phenotype, averaging the effect size of each allele at each locus and thus reducing the variation around the optimum compared to haploid males (for a similar reason, diploid F1 hybrids average the alleles fixed in two parental diploid species and have a higher fitness when those parents have adapted to the same optimum [Barton, 2001; Fraïsse et al., 2016; Simon et al., 2018a]). These results illustrate the complex interplay of evolutionary factors affecting rates of adaptation in haplodiploids vs. diploids.

In Supplementary Materials, we also compare the rates of adaptation in hybrid and parental tetraploid populations (Supplementary Figure 9). As expected, tetraploid parents adapt more slowly than diploid parents because of the high degree of masking of new alleles (new mutation has ¼ effect on the phenotype, [Otto & Whitton, 2000]). However, hybrids again exhibit faster adaptation than parental populations, with the benefits of increased genetic variation hastening adaptation in hybrid tetraploids slightly more than in hybrid diploids (Supplementary Figure 9).

Overall, we find that ploidy plays a relatively minor role in adaptation to the major environmental shifts explored in this paper, whereas hybridization almost always led to substantial increases in the rate of adaptation regardless of model assumptions (see Supplementary Materials for discussion on alternative model assumptions).

## Discussion

Human-induced climate change is increasingly molding the selective environment in which species must either adapt, move, or face extinction (e.g., Aitken et al., 2008; Bell & Gonzalez, 2009). Here we highlight the role that hybridization plays in adaptation to such human-altered environments. Although genetic incompatibilities between two parental populations reduce hybrid fitness when measured in the parental environments, the increased genetic variation that results can hasten adaptation, even when incompatibilities do not depend on the environment. The simulations explored in this paper demonstrate that the increased genetic variation harbored by hybrids dramatically increases the speed of adaptation in novel environments for both haplodiploid and diploid species. Hybrid populations adapt faster than parental populations also in tetraploids, with more trait dimensions and when allowing for population extinction. Using Fisher’s geometric model as a framework, we tracked evolution across the genome for polygenic traits, like thermal tolerance or drought resistance, making these results relevant in light of climate change.

Our simulation results are consistent with increasing evidence of genomic introgression in a variety of species (Edelman & Mallet, 2021; Hedrick, 2013; Suarez-gonzalez et al., 2018) and empirical reports showing that hybrid species can better adapt to environments outside the range experienced by parental populations (Mallet, 2007; Rieseberg et al., 1999; Seehausen, 2004). One of the classic examples of hybridization’s adaptive potential comes from sunflowers, where several species have rapidly originated via homoploid hybrid speciation (Rieseberg et al., 2003). These new hybrid species occur in more extreme habitats where neither parental species is capable of living (Rieseberg, 2006; Ungerer et al., 1998). Importantly, hybridization between *Helianthus annuus* and *H. debilis* was shown to speed up adaptive evolution in a recent eight-year field experiment (Mitchell et al., 2019). Similarly, an experimental study in yeast found that hybrid swarms commonly have a broader environmental range than the average of the parental ranges (Stelkens et al., 2014a), which increased the probability of evolutionary rescue for hybrid populations facing a new environment (Stelkens et al., 2014b). Hybridization has also been inferred to underlie the rapid diversification of Lake Victoria cichlid fish (Meier et al., 2017).In an experimental setting, hybrid beetle populations were able to reach far larger population sizes compared to parents on a challenging host plant (Messina et al., 2020). Hybrid incompatibilities are widespread across hybridizing taxa, but even truly intrinsic incompatibilities can be purged as seen in our simulations. In line with our results, e.g., in *Tigriopus* copepods hybrid lineages were able to purge from incompatibilities in early generations and recover fitness on par with parents (Pereira et al., 2014). However, in some cases hybrid genomes may be inherently unstable due to, e.g., chromosomal rearrangements and TEs, which may reduce the rate of adaptation in hybrids below that of parents as suggested in yeast (Bautista et al., 2021).

In the context of climate adaptation, temperature can favor retention of specific parental haplotypes in hybrids, as shown in laboratory experiments of yeast (Smukowski Heil et al., 2019). In wood ants, alleles from warm and cold adapted parental species segregate in a hybrid population with selection fluctuating between years to favor alleles from warm-adapted species on warm years and alleles from cold-adapted species on cold years (Martin-Roy, et al., 2021). These examples suggest hybridization between cold- and warm-adapted species creates variation that can be accessed by natural selection in real populations. As suggested by empirical examples and our simulations hybridization thus plays more of a creative role than initially thought, allowing adaptation to novel and extreme environments (Mallet, 2007; Rieseberg et al., 1999; Seehausen, 2004).

Although the benefits of hybridization have been realized in selective breeding, speciation studies have focused primarily on the deleterious consequences of hybridization, using hybrid breakdown to find genes acting as barriers for gene flow. For natural populations, the deleterious fitness consequences of hybridization are most relevant to cases where the environment is constant and the species are well adapted. However, at times of rapid ecological changes it is likely that one or both parental species will be unfit in the altered environment, and the relative fitness of hybrids may be on par or even exceed the parental fitness, as found in our simulations.

It is likely that rates of hybridization will increase with on-going climate change, due to redistribution of species and populations, either through natural processes or by human introduction (Chunco, 2014; Ottenburghs, 2021; Scheffers et al., 2016). While we show that elevated rates of hybridization increase the potential for adaptation, the impact on biodiversity is a double-edged sword. On the one hand, hybridization can lead to the loss of species (Owens & Samuk, 2020), particularly when hybrids are better able to adapt and colonize new environments as observed for hybrid grasses between *Spartina alterniflora* and *S. foliosa* in North America (Ainouche et al., 2004). On the other hand, hybridization can provide the genetic variation enabling adaptation of at least some populations in species complexes that would otherwise face extinction.

Our work predicts that hybrid species will be increasingly successful and able to adapt to environmental changes. We may thus see more widespread collapse of related species into hybrid swarms in the future. Examples of such species collapse through hybridization are known in fish, where eutrophication in Alpine lakes has driven extinctions of white fish through hybridization and demographic decline (Vonlanthen et al., 2012) and invasion of signal crayfish in Enos lake in Canada that collapsed two young stickleback species into one (Taylor et al., 2006). With climate change altering habitats around the globe, hybrid populations may increasingly be “winners” in the race to adapt.

Our simulations capture many but not all aspects of adaptation in a changing environment. Our simulations focused on extreme change in the environment, which may accentuate the benefits of increased genetic variation when hybrids first form, relative to a gradually changing environment. The effects of climate change are expected to be strong for species with longer generation times, but more gradual for species with short-generation times. Our simulations do not capture all the complexities of natural populations like spatial structure, multiple fitness optima, assortative mating, on-going gene flow, and interactions with other species in the ecosystem. Nevertheless, we show that faster hybrid adaptation is a common outcome of simulations after a rapid change in the environment, which may have profound consequences for which populations are able to persist in an increasingly human-altered world.

## Supplementary Material

qrad002_suppl_Supplementary_Material

qrad002_suppl_Supplementary_Table

## Data Availability

All simulation code is deposited to Dryad with DOI https://doi.org/10.5061/dryad.vhhmgqnz5.
