# Supplementary material for: On the fast track: hybrids adapt more rapidly than parental populations in a novel environment
Source: Evol Lett. 2023 Mar 1;8(1):128–36. doi: 10.1093/evlett/qrad002 (PMC10871894; doi:10.1093/evlett/qrad002)
Supplement: qrad002_suppl_Supplementary_Material [file qrad002_suppl_supplementary_material.pdf]

Supplementary Material to Kulmuni, Wiley, Otto (2023) On the fast track: Hybrids adapt more rapidly than parental populations in a novel environment

Supplementary Text

In the main text, we made several simplifying assumptions regarding the fitness landscape, ploidy level, nature of genetic incompatibilities, and demography. Here, we extend our simulations to relax these assumptions. Except where noted, we use a smaller set of four scenarios for environmental change, presented in Sup. Fig. 1. We generally find that hybrid populations adapt faster than parental populations following a sudden optimum shift across the supplementary parameter ranges explored. Under certain conditions, we find a reduced hybrid advantage, e.g., when changing the curvature of the fitness function (Sup. Figs. 7 and 8) or when incorporating context-independent hybrid incompatibilities (Sup. Figs. 10 and 11).

1. Altering the genetic assumptions

We first alter the assumption that traits are additive on the phenotypic scale. Specifically, we allow heterozygous mutations to have an effect $h\ t$ for a trait affected by t in homozygotes, where h = 0.8 (dominant mutations) or 0.2 (recessive mutations). The patterns were qualitatively similar in both diploids (Sup. Fig. 2) and haplodiploids (Sup. Fig. 3) to the additive case with h = 0.5 considered in the main text. In this case, the same environmental shifts were considered as in Figures 2 and 3.

We next altered the mean effect size of mutations from λ of 0.05 to 0.8. Compared to the mean value of λ = 0.2 explored in the main text, hybrids adapted faster relative to the parental populations when mutations were smaller on average than when they were larger (Sup. Fig. 4).

2. Changing the fitness landscape

Here, we investigate the effect of relaxing some of the assumptions made about the shape and nature of the fitness landscape by adjusting both the curvature of the fitness function (controlled by the k parameter in Yamaguchi et al. 2020) and the number of phenotypic trait dimensions under selection (controlled by the n parameter).

To explore the effect of increasing trait dimension, we expand the default number of trait dimensions from n=5 in the main text to 10 and 20 in the supplementary simulations, while holding the relative position of environmental optima in the multidimensional trait space constant. For example, parental populations adapting to {2,0,0,0,0} when n=5 instead adapt to {2,0,0,0,0,0,0,0,0,0} when n=10. However, new mutational vectors point randomly in the expanded trait space when n is increased. We find that following the fitness optimum shift at generation 1500, both parental and hybrid populations adapt slower with increasing trait dimensionality (Sup. Fig. 5). This is predicted by the “cost of complexity”, whereby increasing trait dimensionality reduces the probability that new mutations are favourable (Orr, 2000), because with increasing trait dimensions there is an increasing probability that the mutations

point away from the optimum. The reduction in rate of adaptation with increasing *n* is weaker in hybrids, however, compared to parents, meaning the cost of complexity is paid disproportionately by parents. This is because hybrids experience an increase in genetic variation in the period immediately following their creation with increasing *n* (Sup. Fig. 6). This increase in genetic variation is likely caused by an increase in the number of substitutions required to bring the parental phenotypes close to the optimum with larger numbers of dimensions during the initial period of adaptation from generation 0 to 1500.

We next adjusted the curvature of the fitness function, such that fitness falls according to $exp(-x^k)$, where x is the Euclidean distance between an individual's phenotype and the environmental optimum. Specifically, we increase the curvature parameter k from 2 (as used in the main text) to 4 and 6, which results in a flattening of the fitness function close to and far from the optimum (i.e., the fitness landscape becomes more like a tabletop, Sup. Fig. 7). Increasing k makes it more likely that mutations fix that have a minor deleterious effect in conspecifics but have a large deleterious effect (falling off the tabletop) in a heterospecific background (Fraïsse et al., 2016), which may reduce the fitness of hybrids. Consistent with this expectation, we find increasing k results in a reduction in the difference of adaptational speed between parental and hybrid populations (Sup. Fig. 8). Alternatively, with a higher k, populations near the inflection point on the fitness surface experience very strong selection and may not have to adapt far to reach the high fitness plateau, so that the differences in rate of adaptation between parental and hybrid populations is less apparent.

3. Adaptation in tetraploids

In the main text, we examine the rate of hybrid adaptation in diploids and haplodiploids and do not find much systematic differences between them. To further examine the effect of ploidy, we simulated autotetraploid populations with four copies of the genome per individual. Mutations were again set to be additive, with one copy of a mutation in a tetraploid genome having one quarter the effect on the individual phenotype compared to the same mutation with four copies. Recombination is simulated in a similar fashion to diploid simulations, but occurs between two random pairs of the four copies of the genome during the production of each gamete. In general, we find that both hybrid and parental tetraploid populations adapt slower, compared to their diploid counterparts (Sup. Fig. 9). This reflects the stronger masking of new mutations in tetraploids, where the new allele is only one of four gene copies (Otto and Whitton 2000). Nevertheless, the relative speed of hybrid adaptation compared to parental adaptation is similar in both diploids and tetraploids, i.e. hybrids adapt faster than parents in both cases.

4. Incorporating context-independent hybrid incompatibilities

In the main text, we only consider genetic incompatibilities that arise from mismatched alleles that spread during the course of adaptation in the Fisher's geometric model framework. These incompatibilities depend on the genomic background and the environment. However, we might also expect some intrinsic genetic incompatibilities, which cause fitness to decrease no matter the environment (Dobzhansky 1936; Muller 1942). To examine the effect of these

environmentally independent incompatibilities, we introduced pairs of incompatibility loci into our simulations ("BDMI" loci). Following MacPherson et al. (2022), for each randomly located pair of BDMI loci (denoted as **A** and **B,** with ancestral alleles *A* and *B*), we assumed each diploid parental population was fixed for either the *AAbb* or *aaBB* genotype. In haplodiploid populations, we also assumed males were fixed for *Ab* or *aB* genotype in the two populations. We then implemented one of two types of BDMI schemes: 'recessive' or 'dominant'. In the 'recessive' case, for each *aabb* genotype in the diploid hybrid genome or *ab* genotype in the haploid (male) hybrid genome, the individual hybrid fitness calculated under Fisher's geometric model was multiplied by (1-*s*), where *s*=0.05. In the 'dominant' case, this fitness calculated under Fisher's geometric model was multiplied by (1-*s*) for each *aabb*, *Aabb*, *aaBb*, or *AaBb* genotype in diploid hybrids, or for each *ab* genotype in haploid hybrids.

As expected, increasing the number of these BDMI loci resulted in a larger decrease in hybrid fitness immediately following hybridization, both in diploids (Sup. Fig. 10) and in haplodiploids (Sup. Fig. 11). A smaller absolute decrease in fitness was present in scenarios with a larger change in environmental optimum following hybridization ("3D" and "Off"), reflective of the multiplicative deleterious fitness effect of these BDMI loci. In haplodiploid populations, recessive BDMI loci resulted in a comparatively larger drop in hybrid (haploid) male fitness, whereas dominant BDMI loci resulted in a larger drop in hybrid female fitness. These results are consistent with haplodiploid *Formica* ant hybrids, where recessive incompatibilities acting more frequently in hybrid haploid males can cause large-scale differences between male and female genomes (Kulmuni et al., 2010; Kulmuni & Pamilo 2014).

After this initial fitness drop, the speed of adaptation in hybrids depends on the environmental scenario (Sup. Fig. 1). For scenarios where the environmental change was along the same axis as initial adaptation ("Past" and "Future"), hybrids with BDMI loci adapted at a similar rate to those without (Sup. Figs. 10-11). Furthermore, BDMIs were rapidly purged from hybrid genomes (Sup. Fig. 12) and parentally-derived mutations had a stronger tendency to originate from one parental population than the other, leading to a situation where the hybrid genome is composed of a major parent and minor parent (Sup. Fig. 13). In contrast, for scenarios where the environmental change was along a different axis ("Off" and "3D"), hybrids with increasing numbers of BDMI loci adapted at a slower rate, BDMIs were cleared less rapidly, and parentally-derived mutations had a weaker tendency to originate from one parental population over the other. We hypothesize that mixtures of alleles from both parental backgrounds are required to significantly shift the phenotype orthogonally to the original axis of adaptation, which would be beneficial under these scenarios. However, mixing parental backgrounds would conflict with the elimination of intrinsic incompatibilities, which occurs more efficiently with a bias towards a single parental background (Sup. Fig. 14). Nevertheless, hybrids initially adapted faster than parental populations across all scenarios explored.

5. Incorporating population demography

In the main text, we assume that population size remains constant throughout the course of the simulation (e.g., N_{pop}=1500 for diploids). However, we expect that populations would decline in

size when adapting to a novel environment following a major shift in the environmental optimum. This reduced population size can then slow adaptation, due to an increased influence of drift and a reduced influx of new mutations. To examine the effect of demography on our simulations, we simulated explicit population size dynamics with a birth-death model following Yamaguchi et al. (2022). The expected change in the population size between generations *t* and *t*+1 was defined as

$$M(N(t)) = rN(t)(1 - N(t)/K) - N(t)s(t, N(t))$$

where $N(t)$ is the population size at generation *t*, *r* is the intrinsic growth rate, *K* is the carrying capacity (set as the same as population size in simulations without demography), and $s(t, N(t))$is 1 minus the mean population fitness at time *t*. Similarly, the variance in the change in population size between generations *t* and *t+1* was defined as

$$V(N(t)) = rN(t)(1 - N(t)/K) + N(t)(s(t, N(t)) + 2\beta)$$

where β is additional variance in population size change present when the population is at carrying capacity (set as 0.1). Then, in simulations the actual population size change between each generation was set as

$$\Delta N = M(N(t)) + \sqrt{V(N(t)}\Delta\omega)$$

where $\Delta\omega$ is a random draw from a standard normal distribution.

We were also interested in determining the difference between hybrids and parents in extinction probability following environmental optima shift. To this end, we scaled the original scenarios depicted in Sup. Fig. 1 so that all populations experienced an optimum shift of distance 2. This was done because with smaller optimum shifts very few populations went extinct. Under the 'past' scenario, the optimum shifted to {0,0,0,0,0} at generation 1500. Similarly, under the 'future' scenario, the optimum shifted to {4,0,0,0,0} at generation 1500. Under the 'off' scenario the parental populations initially adapted to {1.414,0,0,0,0}, and then the optimum shifted to {0,1.414,0,0,0} at generation 1500. Similarly, under the '3D' scenario parental populations initially adapted to {1.414,0,0,0,0} and {0,1.414,0,0,0}, respectively, and then the optimum shifted to {0,0,1.414,0,0} at generation 1500.

To ensure a suitable number of replicates, for each combination of environmental scenario, intrinsic growth rate, and ploidy level we ran replicate simulations until 200 simulations were attained with at least one population that survived until generation 1800 (either the hybrid population or one of the two simulated parental populations). The resulting numbers of replicate populations for each parameter combination are stored in a supplementary excel file.

As in the simulations with a fixed population size explored in the main text, hybrids adapted faster than the parental populations, regardless of environmental scenario, for both diploids

(Sup. Fig. 14) and haplodiploids (Sup. Fig. 15) when population demography was taken into account.

Different patterns were seen, however, in the rate of extinction of hybrids versus parental populations depending on the intrinsic growth rate, *r* (Sup. Fig. 16). As expected, all populations are more likely to go extinct when growth rates are smaller, yet hybrid populations are more likely to persist under these circumstances than parental populations. Specifically, when *r* = 1, the per capita growth rate becomes negative after the shift in optimum by 2 units, even in the absence of competition ($M(N(t))/N(t) \approx -0.0017$ for $N(t) \approx 0$), causing the populations to decline towards extinction (Sup. Fig. 16). In this case, hybrids are more likely to experience evolutionary rescue, because of their greater access to genetic variance (Sup. Fig. 17) and faster rate of adaptation (Sup. Figs. 14-15). By contrast, with higher intrinsic growth rates, populations are able to grow once the populations have declined to the point that they are released from competition (e.g., ($M(N(t))/N(t) \approx 0.2$ for $N(t) \approx 0$ when *r* = 1.2)). With this quick release from competition, populations largely retain access to the genetic variation that was present before the environmental shift, allowing rapid adaptation and recovery of the population (Sup. Fig. 18). While this occurs for both parental and hybrid populations, the initial hybrid breakdown causes a slightly more severe population bottleneck (see inset panel of Sup. Fig. 17), placing hybrids at greater risk of extinction. Thus, we find a counterintuitive result that when extinction risk is high (here, *r* = 1), hybrids are more likely to persist than parental populations due to evolutionary rescue, but when the environmental change is moderate and extinction risk lower (here, *r* = 1.2 or 1.4), hybrids become more prone to extinction than parental populations due to the combined impact of hybrid breakdown and a changing optimum.

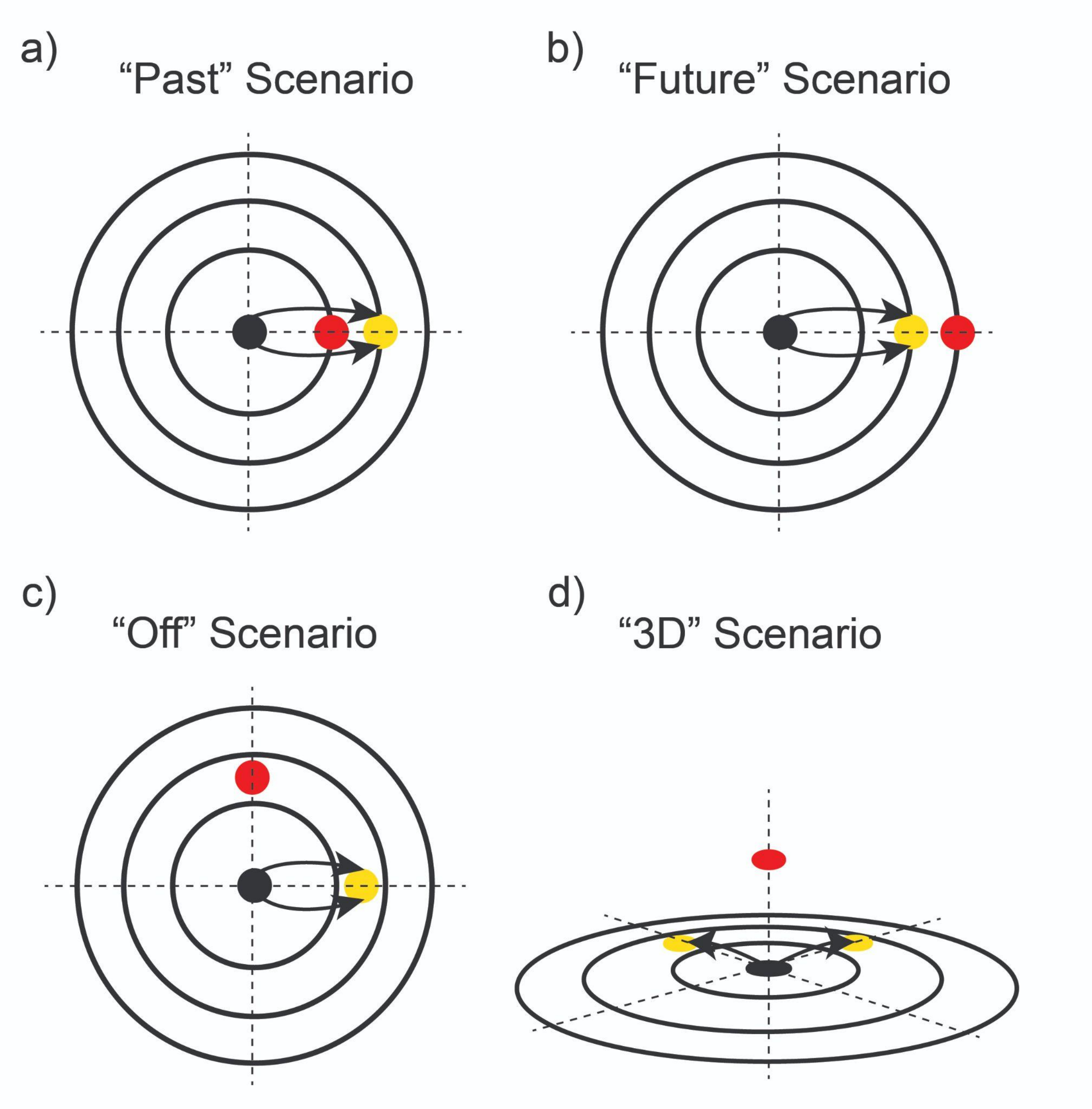

Sup. Fig. 1: Simulation scenarios used for all supplementary simulations except those examining the effect of demography and those presented in Sup. Fig. 2-3. All parental populations start the simulation being adapted to {0,0,0,0,0}. A) In the "past" scenario, the parental populations adapt to {2,0,0,0,0} and the new fitness optimum after hybridization is {1,0,0,0,0} for all populations. B) In the "future" scenario, the parental populations adapt to {2,0,0,0,0} and the new fitness optimum after hybridization is {3,0,0,0,0} for all populations. C) In the "off" scenario, the parental populations adapt to {1.5,0,0,0,0} and the new fitness optimum after hybridization is {0,1.5,0,0,0} for all populations. D) In the "3D" scenario, the two parental populations adapt to {1.5,0,0,0,0} and {0,1.5,0,0,0} respectively, and the new fitness optimum after hybridization is {0,0,1.5,0,0} for all populations.

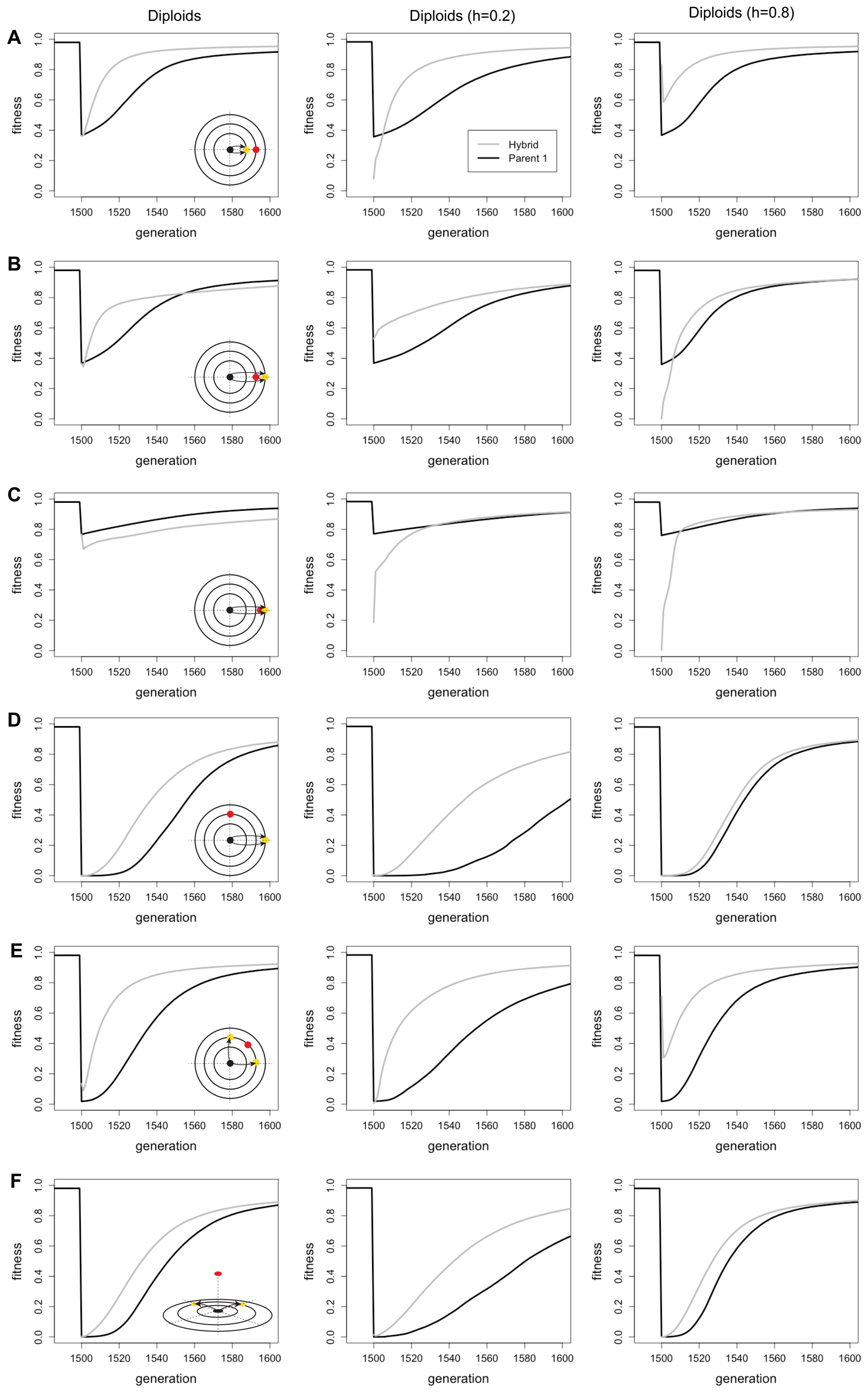

Sup. Fig. 2. Diploid simulations with dominance on the phenotypic scale. Simulations are the same as the diploid case explored in Figures 2 and 3, but mutations are either codominant (first column, as previously illustrated in Figures 2 and 3), partially recessive (second column, *h*=0.2), or partially dominant (third column, *h*=0.8).

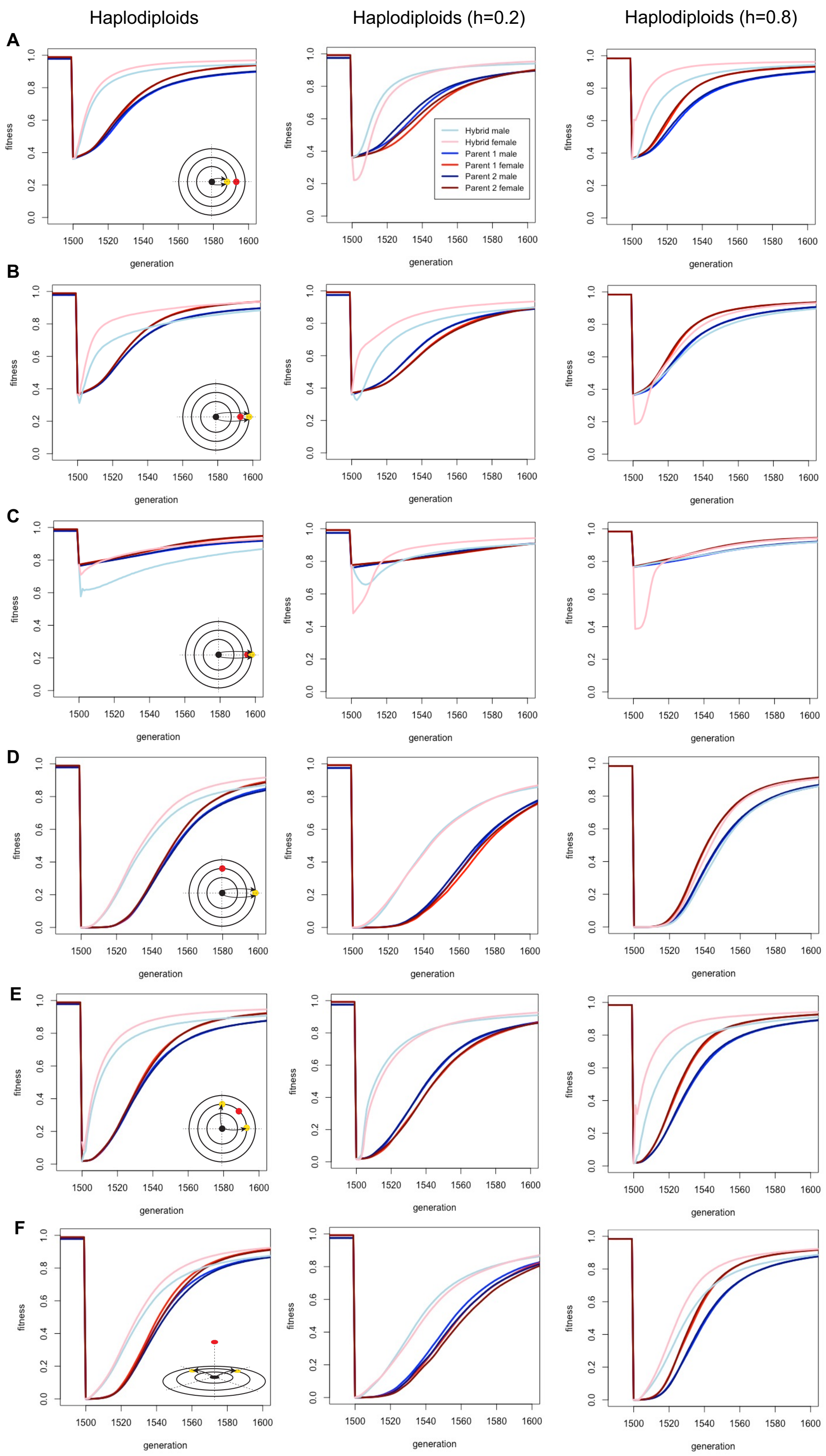

Sup. Fig. 3. Haplodiploid simulations with dominance on the phenotypic scale. Simulations are the same as the haplodiploid cases explored in Figures 2 and 3, but mutations are either codominant (first column, identical to those illustrated in Figures 2 and 3), partially recessive (second column, h=0.2), or partially dominant (third column, h=0.8).

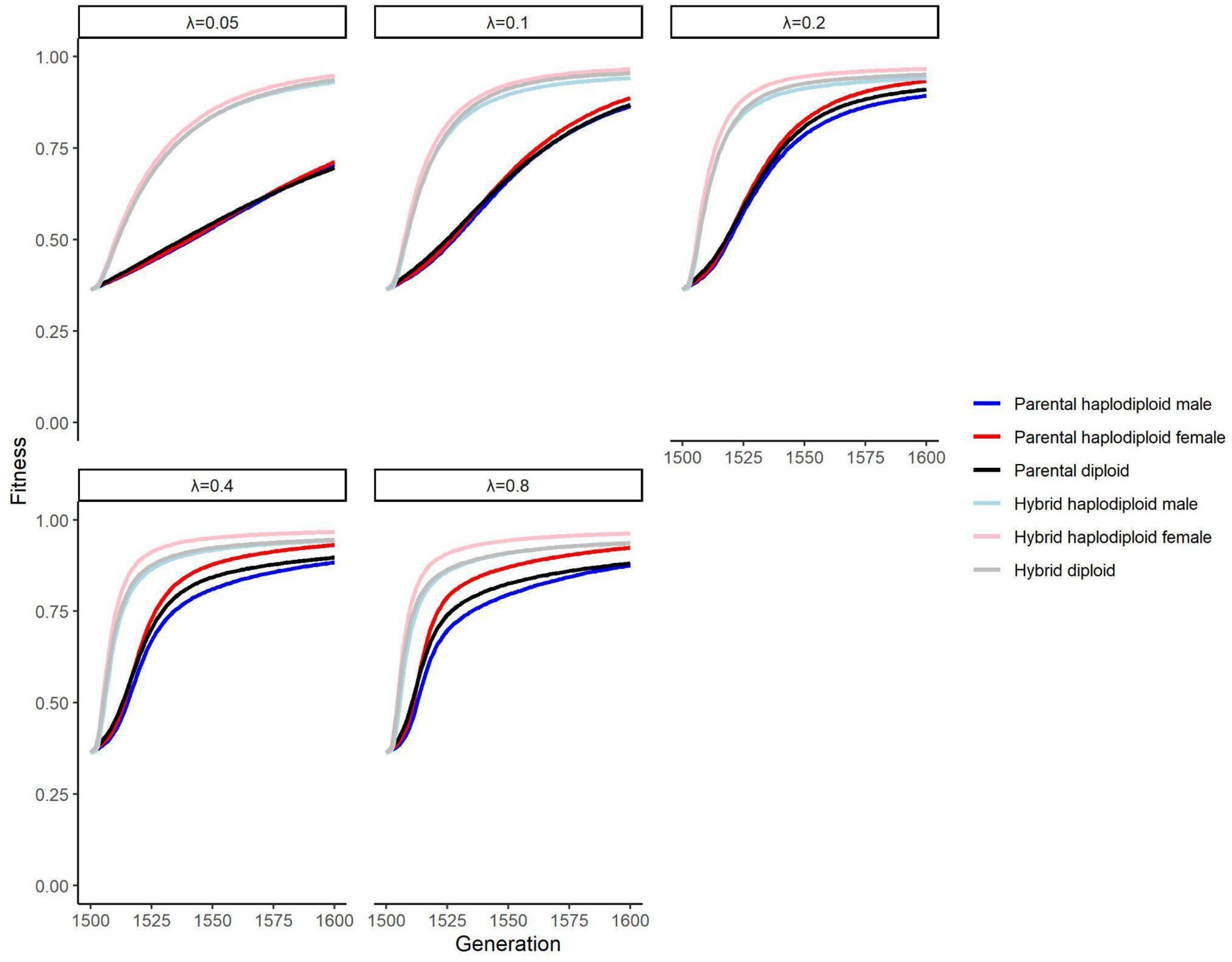

Sup. Fig. 4. Adaptation in hybrid and parental populations when mutations have different effect sizes. Mean fitness in a new environment is shown over time in hybrid (transparent) and parental (dark) haplodiploid and diploid populations with panels showing increasing average effects of mutations (λ). Simulations are otherwise identical to Fig. 2A, with the optimum initially moved from {0,0,0,0,0} to {1,0,0,0,0} at generation 0, followed by a further environmental change to {2,0,0,0,0} at generation 1500, at which point the rate of adaptation is compared for hybrid and parental populations. Fitness values for parental haplodiploid males, parental haplodiploid females, and parental diploids are averages between parental populations.

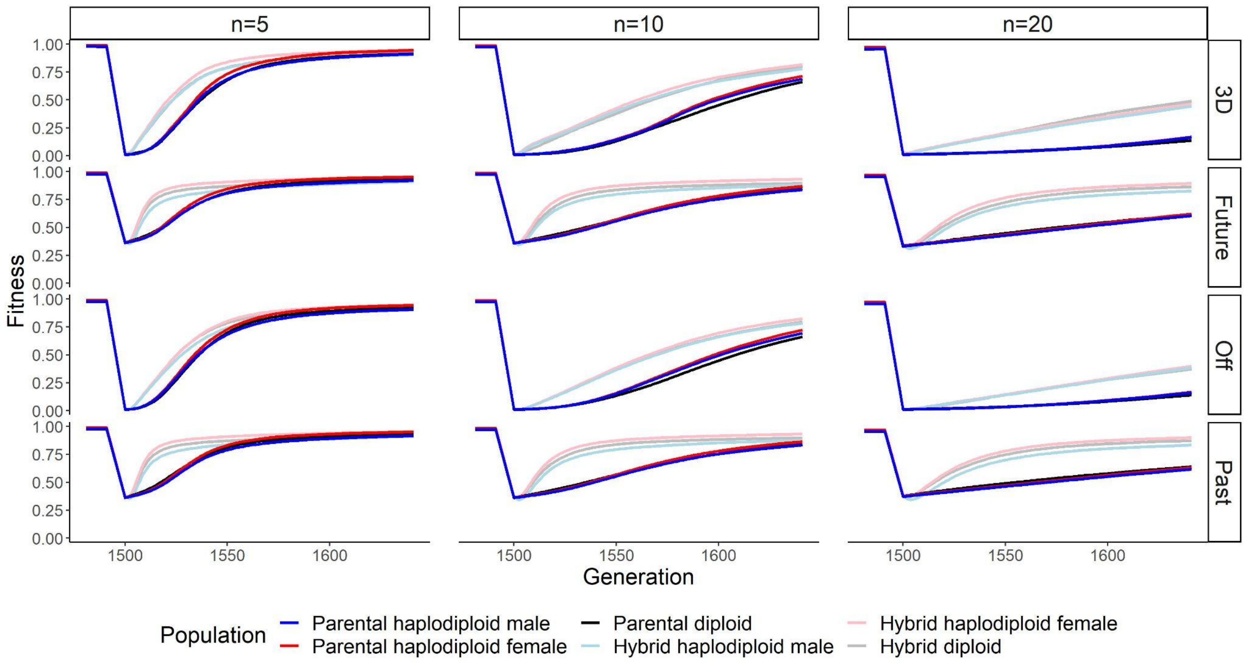

Sup. Fig. 5: Adaptation in hybrids and parental populations with different numbers of available trait dimensions (*n*). The adaptational scenarios indicated on the right-hand box labels are illustrated in Sup. Fig. 1, with additional dimensions added to optima to reflect the appropriate number of available trait dimensions. For example, in the "Past" scenario parental populations initially adapt to an environmental optimum of {2,0,0,0,0} when n=5, and {2,0,0,0,0,0,0,0,0,0} when *n*=10. Fitness values are averaged over 100 replicate simulations for all combinations of *n* values, scenarios, ploidies, and populations.

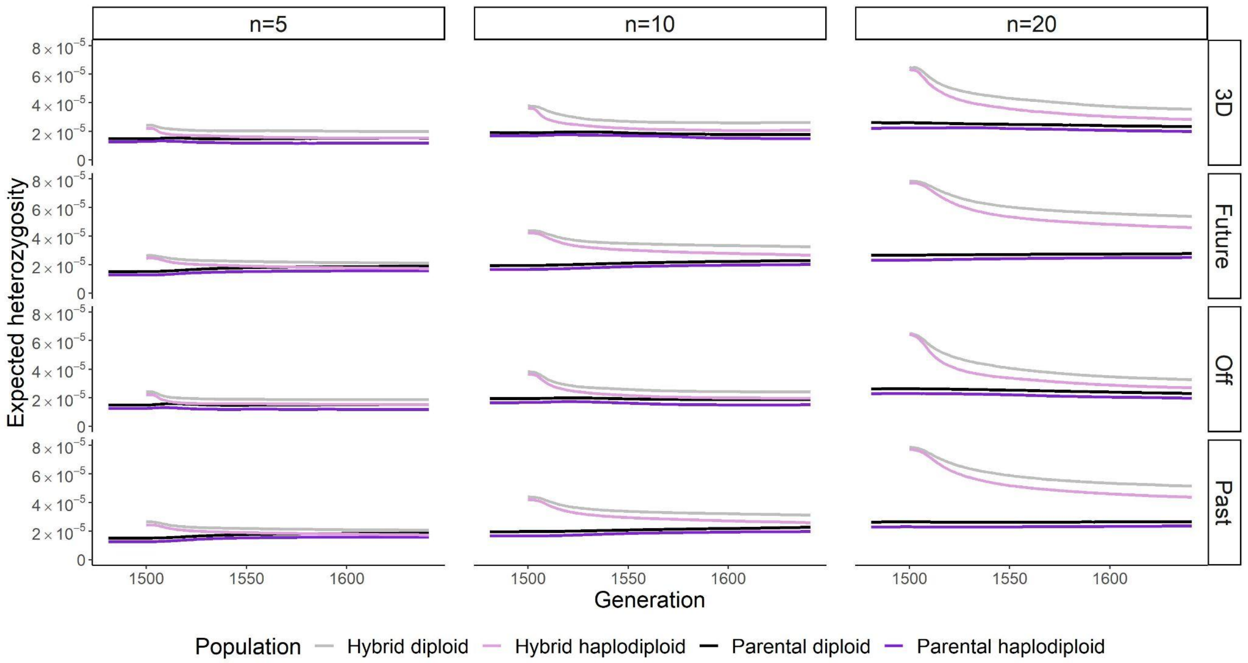

Sup. Fig. 6: The expected heterozygosity (π) in hybrid and parental haplodiploid and diploid populations with different numbers of available trait dimensions (n). The adaptational scenarios indicated on the right-hand box labels are illustrated in Sup. Fig. 1. The expected heterozygosity is calculated as the probability that two randomly chosen alleles from the population are different at a site, averaged over all one million sites in the genome. Expected heterozygosity values are averaged over 100 replicate simulations for all combinations of n values, scenarios, ploidies, and populations.

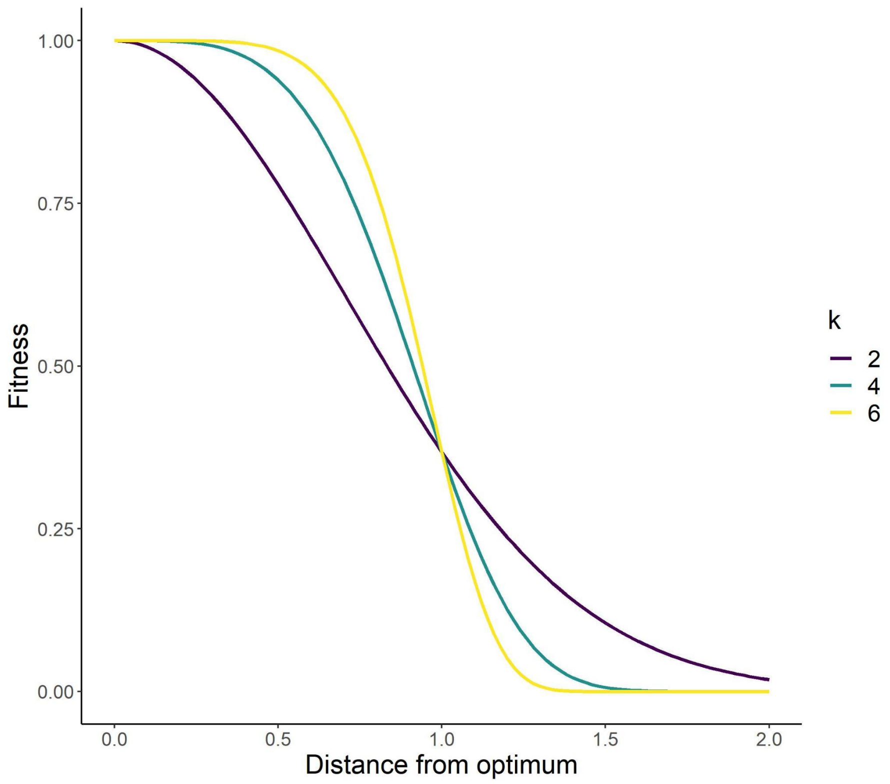

Sup. Fig. 7: The shape of the fitness function with differing curvature parameter values (*k*). Individual fitness is defined as *exp*(-x^k), where *x* is the Euclidean distance of an individual's phenotype from the environmental optimum.

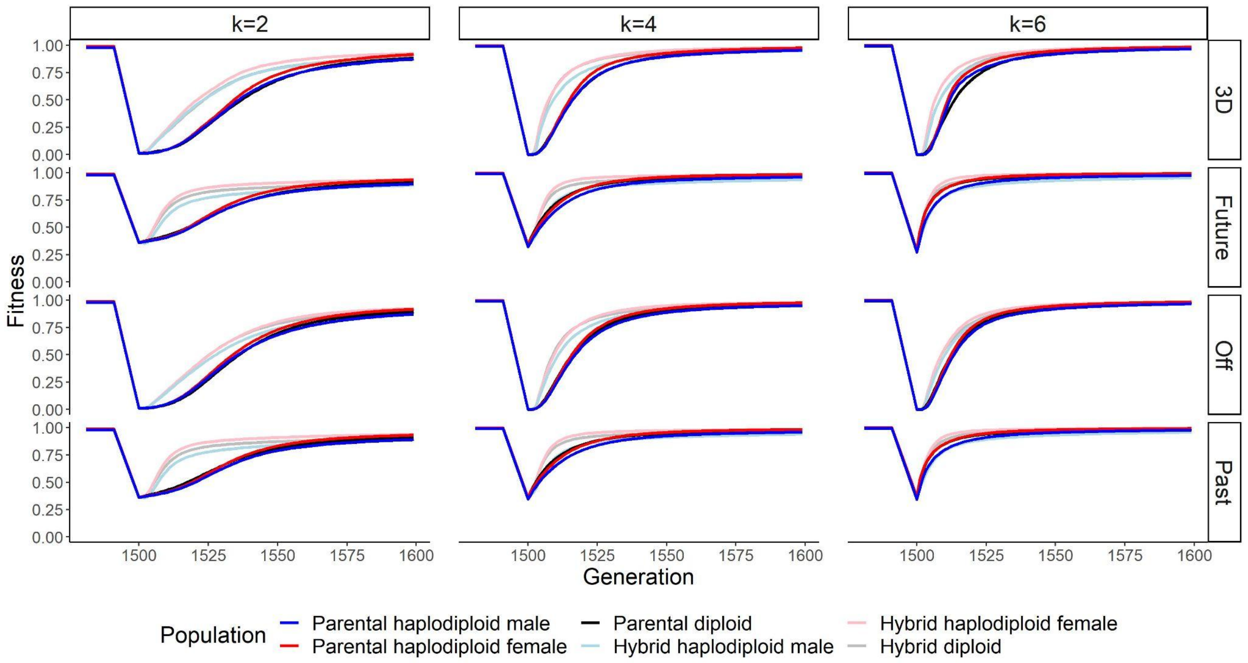

Sup. Fig. 8: Adaptation into a new environment in hybrid and parental haplodiploid and diploid populations with different values of the fitness function curvature parameter (k). Differences in the shape of the fitness function for different values of k are illustrated in Sup. Fig. 7. The adaptational scenarios indicated on the right-hand box labels are illustrated in Sup. Fig. 1. Fitness values are averaged over 100 replicate simulations for all combinations of k values, scenarios, ploidies, and populations.

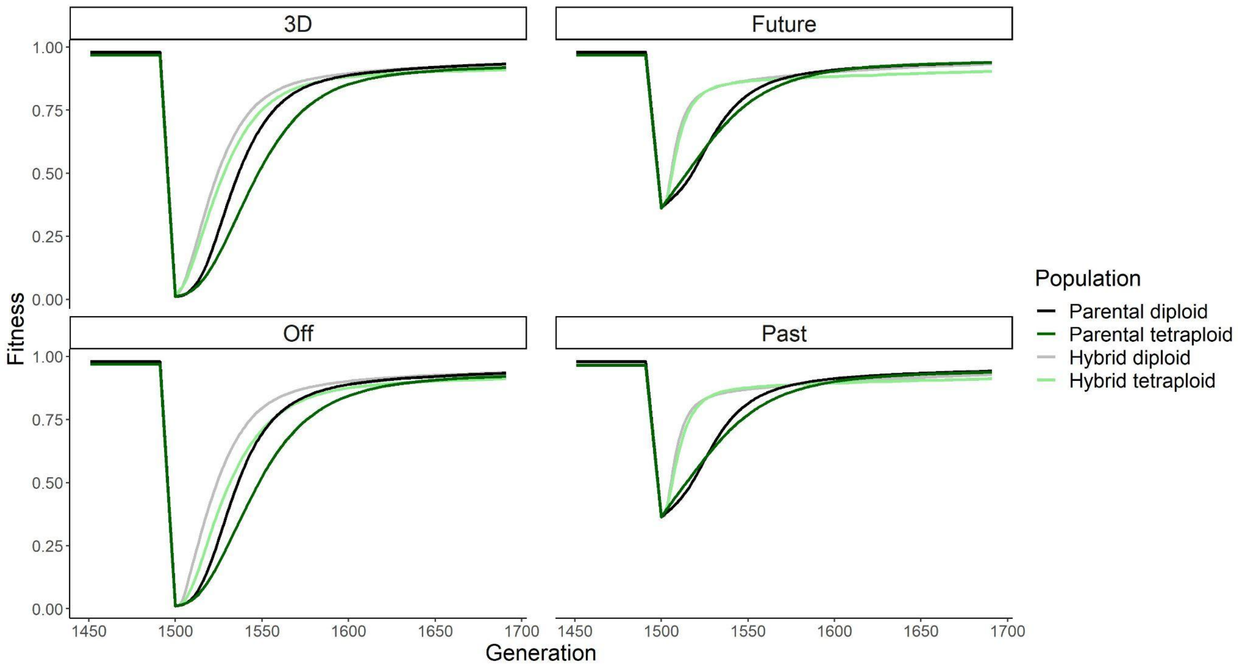

Sup. Fig. 9: Adaptation into a new environment in hybrid and parental diploid and tetraploid populations. The adaptational scenarios in each box label are illustrated in Sup. Fig. 1. Fitness values are averaged over 100 replicate simulations for all combinations of scenarios, ploidies, and populations.

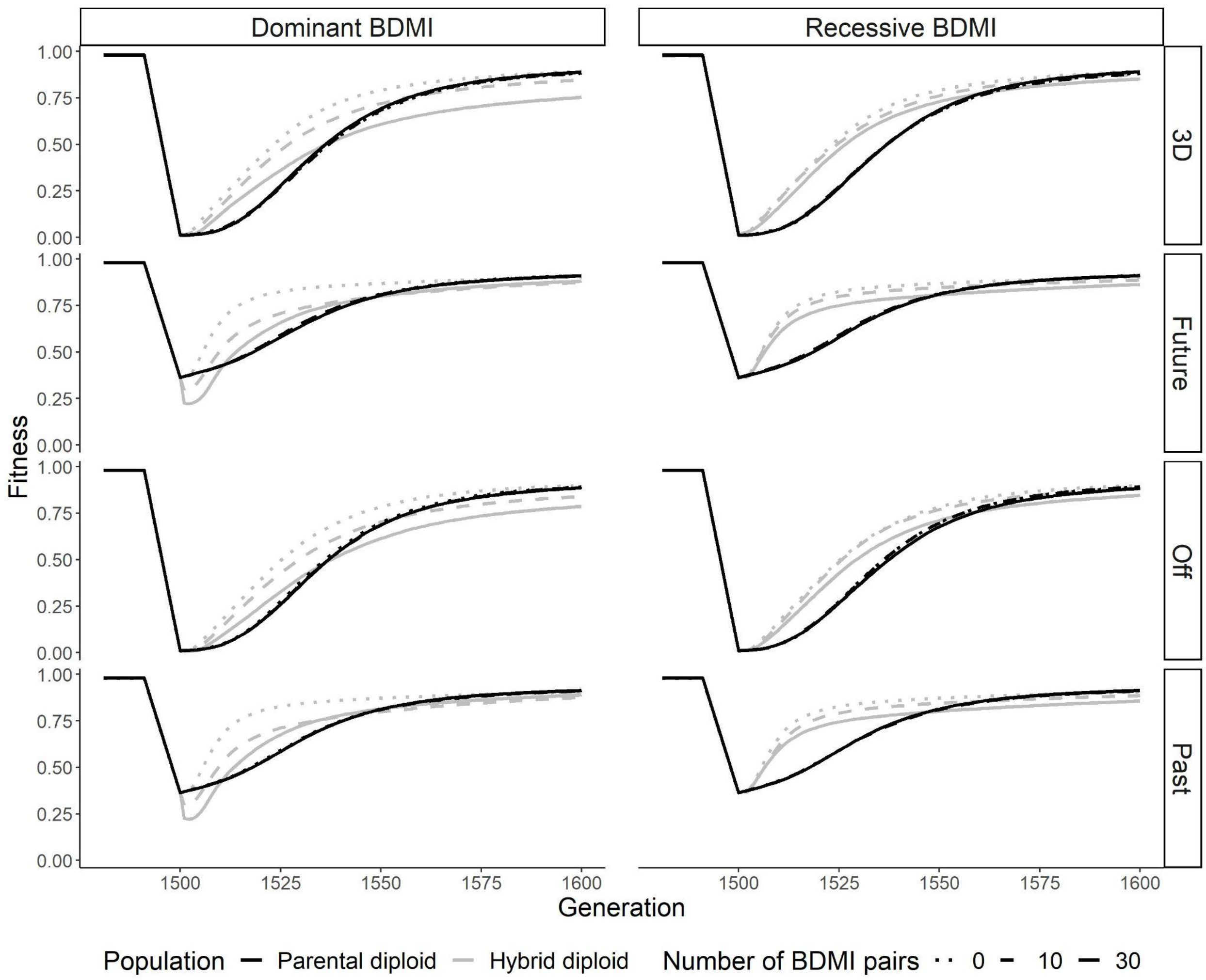

Sup. Fig. 10: Adaptation into a new environment in diploid populations with differing numbers and types of BDMI loci pairs. The adaptational scenarios in each right hand box label are illustrated in Sup. Fig. 1. Fitness values are averaged over 100 replicate simulations for all combinations of scenarios, BDMI pair numbers and types, and populations.

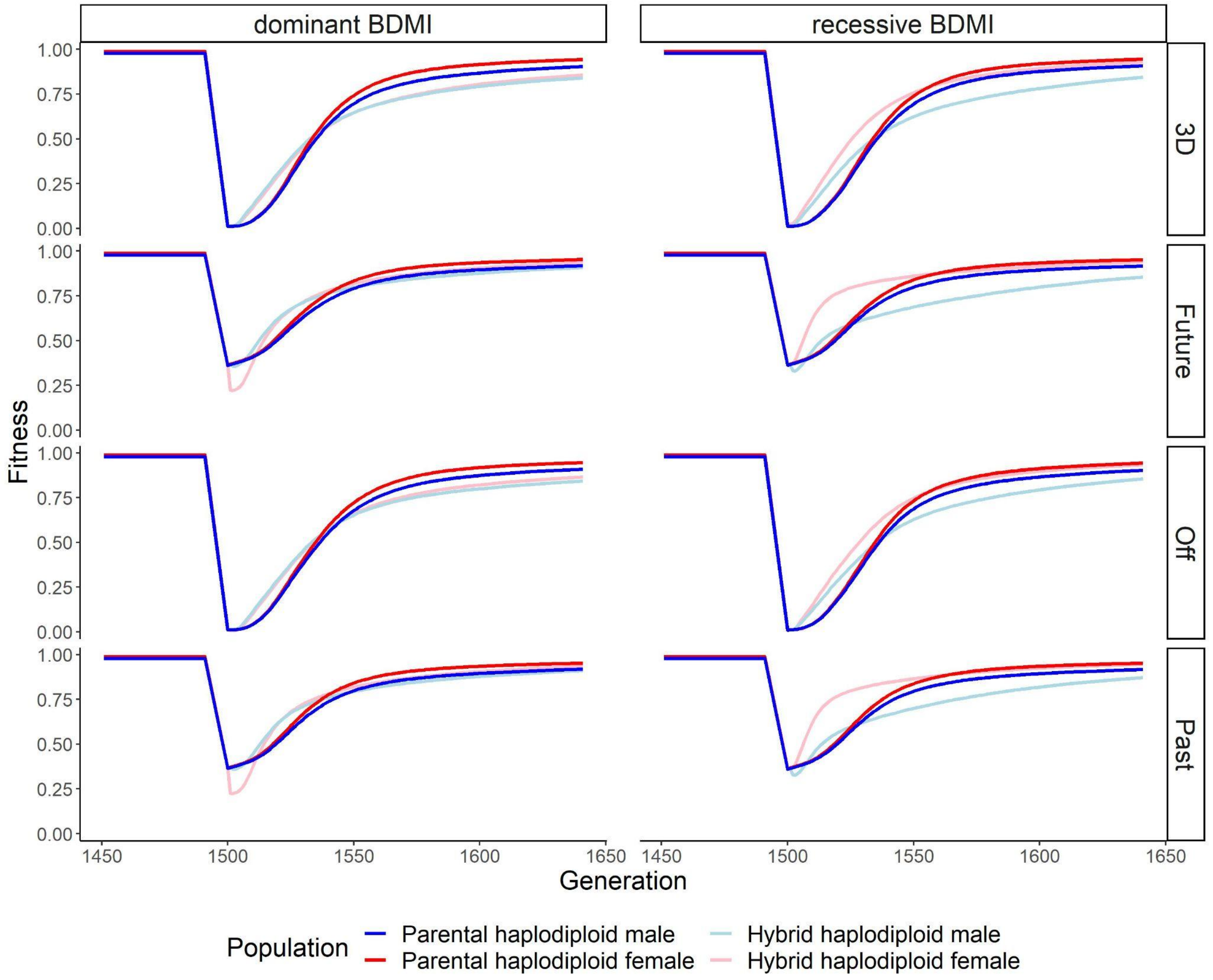

Sup. Fig. 11: Adaptation into a new environment in haplodiploid populations with differing types of BDMI loci pairs. All simulations were conducted with 30 pairs of BDMI loci. The adaptational scenarios in each right hand box label are illustrated in Sup. Fig. 1. Fitness values are averaged over 100 replicate simulations for all combinations of scenarios and populations.

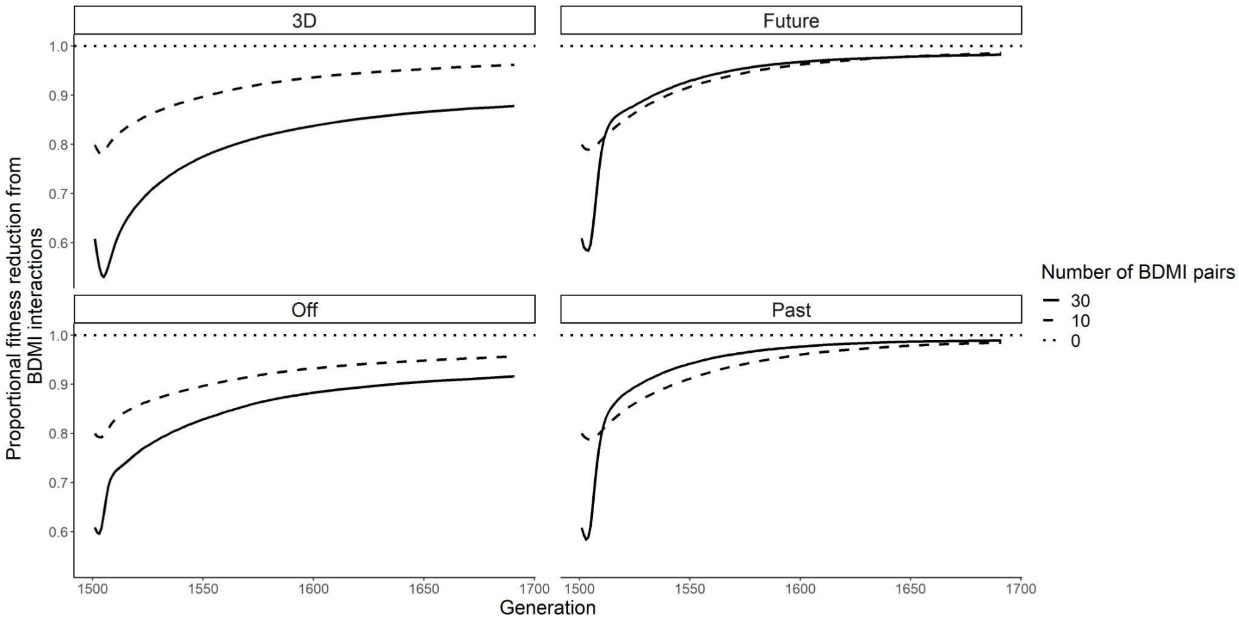

Sup. Fig. 12: The average deleterious effect of BDMI interactions on hybrid fitness for different scenarios and numbers of dominant BDMI loci pairs. For each pair of BDMI loci, an incompatible genotype reduces fitness multiplicatively by (1-*s*), where *s*=0.05. The y-axis displays the total multiplicative deleterious effect of all incompatible BDMI loci, averaged over 100 replicate simulations (for comparison, multiplicative deleterious effect is one with no BDMI loci). Only values from diploid simulations with dominant BDMI loci are shown. The adaptational scenarios indicated on each box label are illustrated in Sup. Fig. 1.

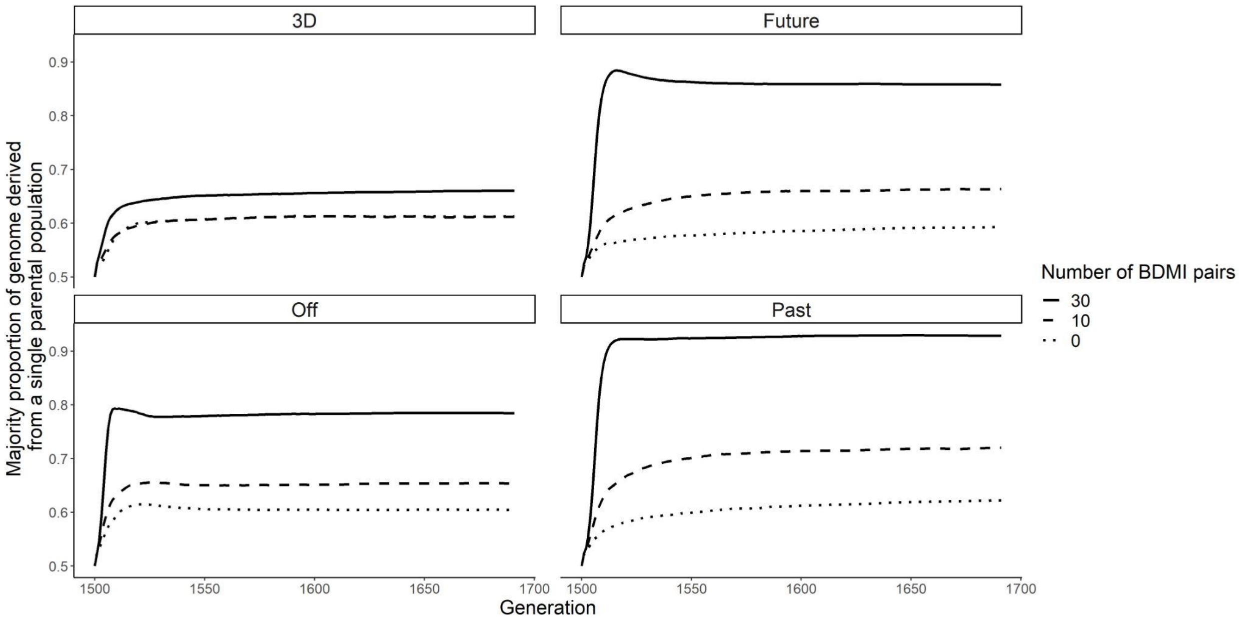

Sup. Fig. 13: Asymmetric contributions from the parental genomes in hybrids rises with the number of BDMI loci. To calculate this measure, the average proportion of alleles that originated from each parental population was calculated across the genome for all hybrid individuals. The fraction coming from the parent comprising the larger fraction of the genome is then shown over time. A larger value thus reflects a bias for hybrid individuals to contain more alleles derived from one parental population compared to the other. The graphed values are averages across 100 replicate simulations for scenarios considered and only show diploid simulations with dominant BDMI loci. The adaptational scenarios indicated on each box label are illustrated in Sup. Fig. 1.

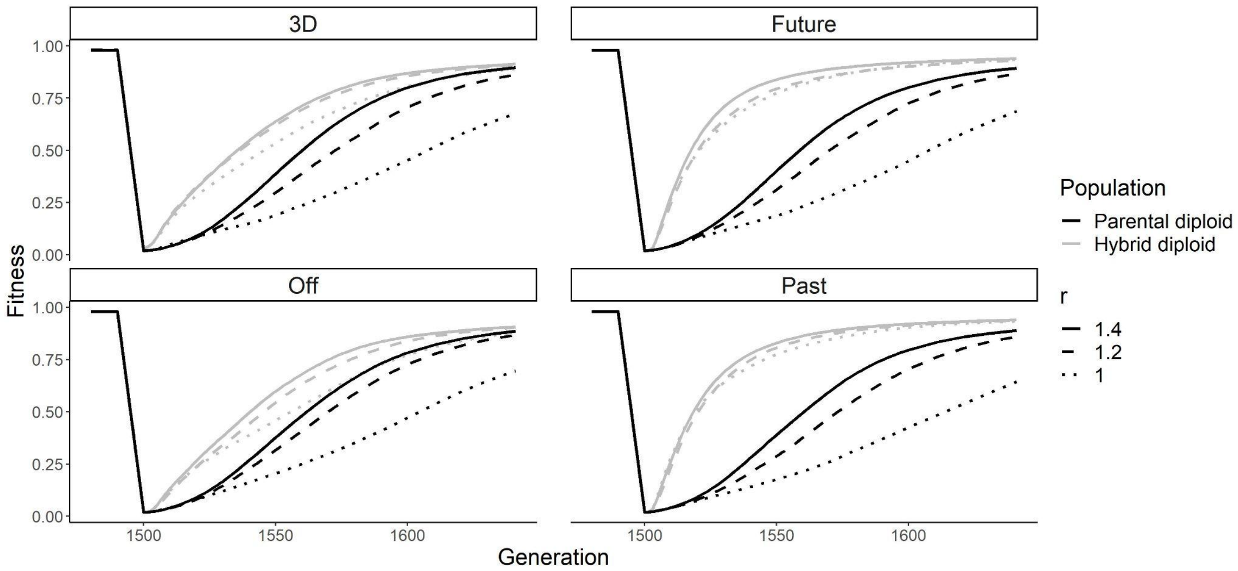

Sup. Fig. 14: Adaptation into a new environment in diploid populations with simulated demographic dynamics and different intrinsic growth rates (*r*). Values are averages from 200 replicate simulations where at least one parental or hybrid population did not go extinct before generation 1800, and only values from populations that did not go extinct are plotted. The exact number of replicates for each parameter combination are available in a Supplementary table 1. The adaptational scenarios indicated on box labels are illustrated in Sup. Fig. 1.

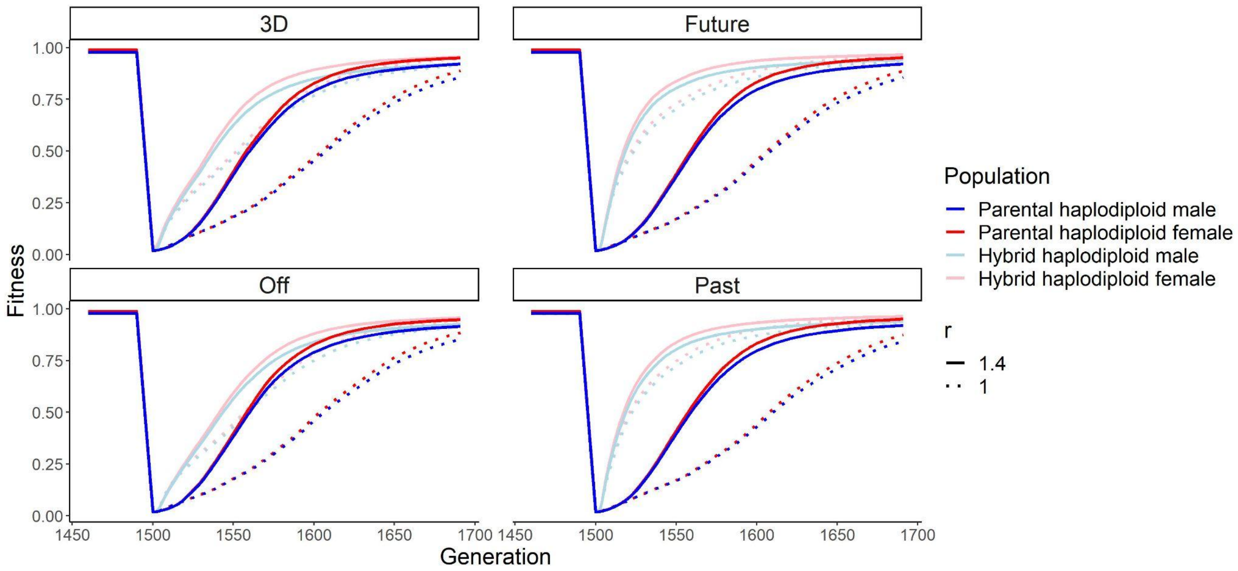

Sup. Fig. 15: Adaptation into a new environment in haplodiploid populations with simulated demographic dynamics and different intrinsic growth rates (*r*). Identical to Sup. Fig. 14, except for haplodiploid populations.

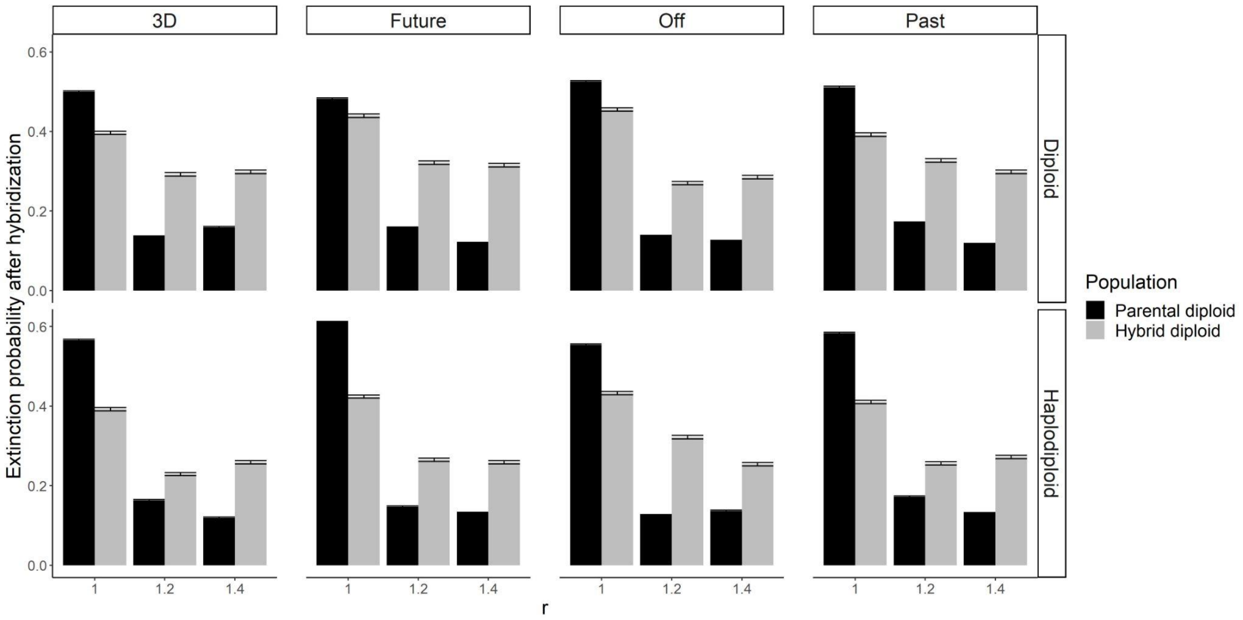

Sup. Fig. 16: Extinction probabilities after hybridization for diploid and haplodiploid populations with simulated demographic dynamics and different intrinsic growth rates (*r*). Values are averages from 200 replicate simulations where at least one parental or hybrid population did not go extinct before generation 1800. The exact number of replicates for each parameter combination are available in Supplementary table 1. The adaptational scenarios indicated on box labels are illustrated in Sup. Fig. 1.

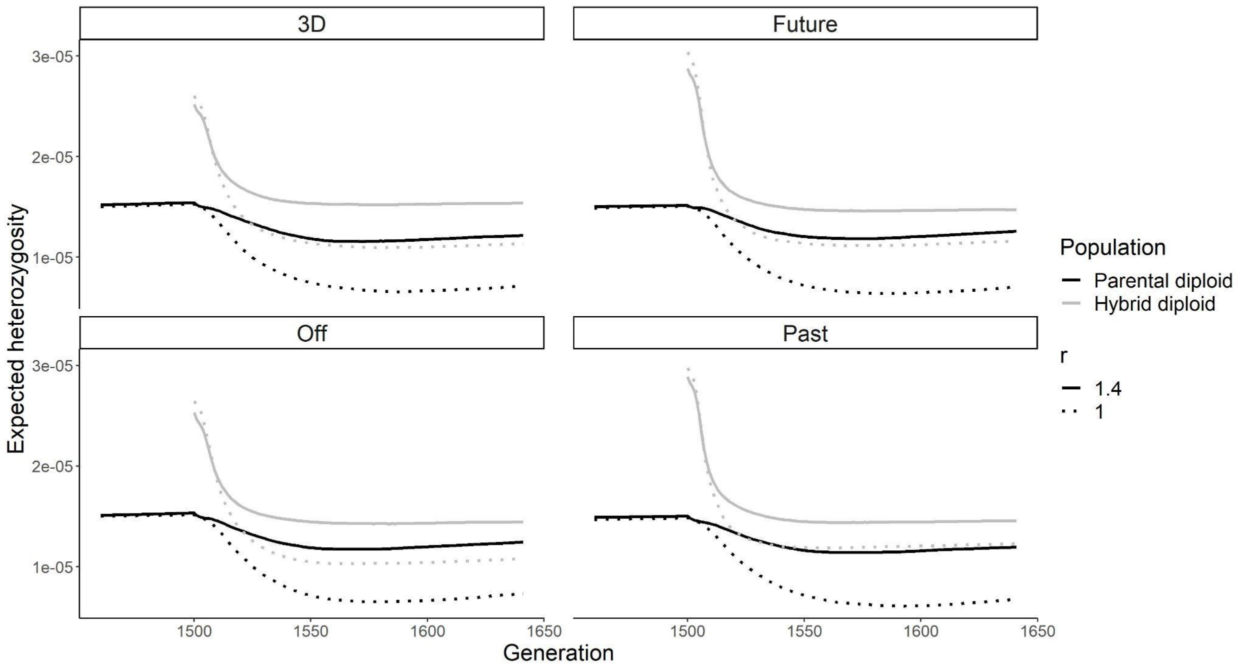

Sup. Fig. 17: The expected heterozygosity (*π*) in hybrid and parental diploid populations with simulated demographic dynamics and different intrinsic growth rates (*r*). Values are averages from 200 replicate simulations where at least one parental or hybrid population did not go extinct before generation 1800, and only values from populations that did not go extinct are plotted. The exact number of replicates for each parameter combination are available in Supplementary table 1. The adaptational scenarios indicated on the box labels are illustrated in Sup. Fig. 1. The expected heterozygosity is calculated as the probability that two randomly chosen alleles from the population are different at a site, averaged over all one million sites in the genome.

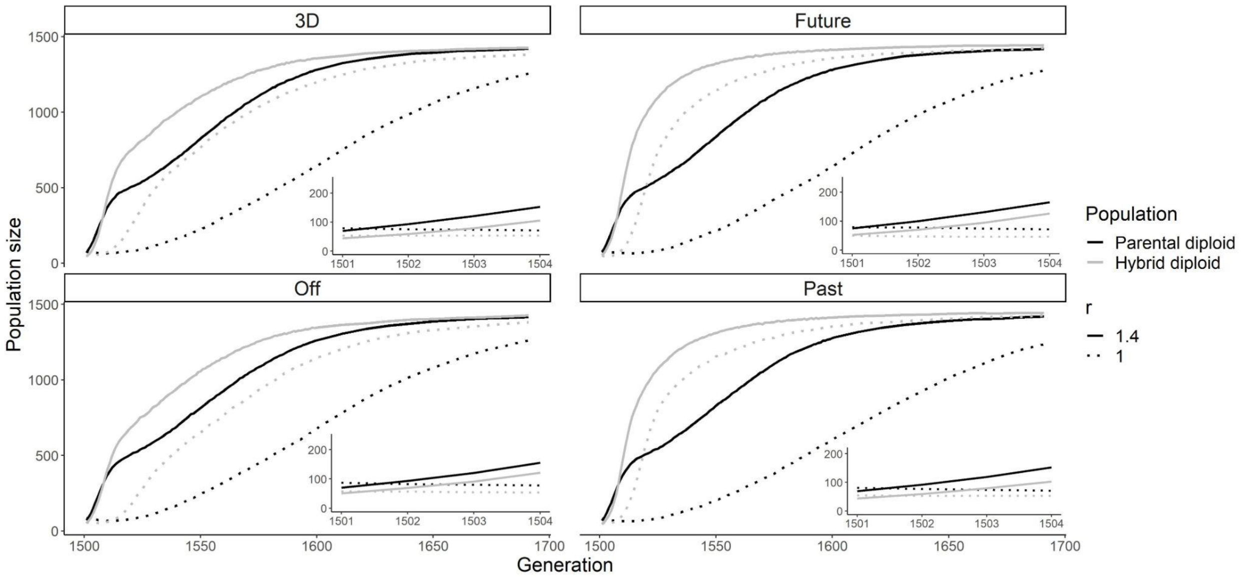

Sup. Fig. 18: The population size in hybrid and parental diploid populations with simulated demographic dynamics and different intrinsic growth rates (*r*). Values are averages from 200 replicate simulations where at least one parental or hybrid population did not go extinct before generation 1800, and only values from populations that did not go extinct are plotted. Values are averages from 200 replicate simulations where at least one parental or hybrid population did not go extinct before generation 1800. The adaptational scenarios indicated on the box labels are illustrated in Sup. Fig. 1.

Sup. Table 1: The number of simulated populations for each ploidy, r value, adaptational scenario, and population (parental or hybrid) used to generate Supplementary figures 14, 15, 17, and 18. Of these, the number of populations that survived to generation 1800 or went extinct is also given, and these values are used to generate Supplementary figure 16. For each parameter combination, replicate simulations were run until 200 simulations were achieved where at least one parental or hybrid population did not go extinct, resulting in variable replicate numbers for each population. Adaptational scenarios are illustrated in Supplementary figure 1.

Ploidy	r	Adaptational Scenario	Population	Total number of simulated populations	Number of populations extinct before generation 1800	Number of populations that survived up to generation 1800
Diploid	1	3D	parental	444	222	222
Diploid	1	3D	hybrid	222	88	134
Diploid	1	Future	parental	446	215	231
Diploid	1	Future	hybrid	223	98	125
Diploid	1	Off	parental	466	245	221
Diploid	1	Off	hybrid	233	106	127
Diploid	1	Past	parental	444	227	217
Diploid	1	Past	hybrid	222	87	135
Diploid	1,2	3D	parental	404	55	349
Diploid	1,2	3D	hybrid	202	59	143
Diploid	1,2	Future	parental	404	64	340
Diploid	1,2	Future	hybrid	202	65	137
Diploid	1,2	Off	parental	400	55	345
Diploid	1,2	Off	hybrid	200	54	146
Diploid	1,2	Past	parental	404	69	335
Diploid	1,2	Past	hybrid	202	66	136
Diploid	1,4	3D	parental	402	64	338
Diploid	1,4	3D	hybrid	201	60	141
Diploid	1,4	Future	parental	400	48	352
Diploid	1,4	Future	hybrid	200	63	137
Diploid	1,4	Off	parental	400	50	350
Diploid	1,4	Off	hybrid	200	57	143
Diploid	1,4	Past	parental	402	47	355
Diploid	1,4	Past	hybrid	201	60	141
Haplodiploid	1	3D	parental	454	257	197
Haplodiploid	1	3D	hybrid	227	89	138
Haplodiploid	1	Future	parental	486	297	189

Haplodiploid	1	Future	hybrid	243	103	140
Haplodiploid	1	Off	parental	462	256	206
Haplodiploid	1	Off	hybrid	231	100	131
Haplodiploid	1	Past	parental	468	273	195
Haplodiploid	1	Past	hybrid	234	96	138
Haplodiploid	1,2	3D	parental	410	67	343
Haplodiploid	1,2	3D	hybrid	205	47	158
Haplodiploid	1,2	Future	parental	400	59	341
Haplodiploid	1,2	Future	hybrid	200	53	147
Haplodiploid	1,2	Off	parental	404	51	353
Haplodiploid	1,2	Off	hybrid	202	65	137
Haplodiploid	1,2	Past	parental	406	70	336
Haplodiploid	1,2	Past	hybrid	203	52	151
Haplodiploid	1,4	3D	parental	402	48	354
Haplodiploid	1,4	3D	hybrid	201	52	149
Haplodiploid	1,4	Future	parental	402	53	349
Haplodiploid	1,4	Future	hybrid	201	52	149
Haplodiploid	1,4	Off	parental	402	55	347
Haplodiploid	1,4	Off	hybrid	201	51	150
Haplodiploid	1,4	Past	parental	404	53	351
Haplodiploid	1,4	Past	hybrid	202	55	147

Supplementary References

Dobzhansky T. 1936. Studies on hybrid sterility. II. Localization of sterility factors in Drosophila pseudoobscura hybrids. *Genetics,* 21:113-135.

Fraïsse, C., Gunnarsson, P. A., Roze, D., Bierne, N., & Welch, J. J. (2016). The genetics of speciation: insights from Fisher's geometric model. *Evolution*, *70:*1450-1464.

Kulmuni, J., Seifert, B., & Pamilo, P. (2010). Segregation distortion causes large-scale differences between male and female genomes in hybrid ants. *Proceedings of the National Academy of Sciences, 107:* 7371-7376.

Kulmuni, J., & Pamilo, P. (2014). Introgression in hybrid ants is favored in females but selected against in males. *Proceedings of the National Academy of Sciences*, 111:12805-12810.

MacPherson, A., Wang, S., Yamaguchi, R., Rieseberg, L.H, Otto, S.P. (2022). Parental population range expansion before secondary contact promotes heterosis. *The American Naturalist,* 200:1, E1-E15

Muller HJ. 1942. Isolating mechanisms, evolution, and temperature. *Biol. Symp*. 6:71-125.

Orr, H. A. (2000). Adaptation and the cost of complexity. *Evolution*, *54:*13-20.

Otto, S. P., & Whitton, J. (2000). Polyploid incidence and evolution. Annual Review of Genetics, 34(1), 401-437

Yamaguchi, R., Wiley, B., & Otto, S. P. (2022). The phoenix hypothesis of speciation. *Proceedings of the Royal Society B*, *289*(1987), 20221186.
